# A pandemic of delirium: an updated systematic review and meta-analysis of occurrence of delirium in older adults with COVID-19

**DOI:** 10.1007/s41999-023-00906-7

**Published:** 2024-03-18

**Authors:** Maria Beatrice Zazzara, Alice Margherita Ornago, Camilla Cocchi, Elisabetta Serafini, Giuseppe Bellelli, Graziano Onder

**Affiliations:** 1 Fondazione Policlinico Universitario Agostino Gemelli IRCCS, Largo Agostino Gemelli 8, 00168 Rome, Italy; 2 School of Medicine and Surgery, University of Milano-Bicocca, Milan, Italy; 3 Department of Aging, Orthopaedics and Rheumatological Sciences, Università Cattolica del Sacro Cuore, Rome, Italy; 4 Acute Geriatrics Unit, Fondazione IRCCS San Gerardo dei Tintori, Monza, Italy

**Keywords:** Older adults, Delirium, COVID-19, Frailty, SARS-CoV-2 infection

## Abstract

**Purpose:**

Delirium has been recognized as an atypical presenting feature of COVID-19 in older adults and is independently associated with mortality. We aimed to perform an updated systematic review of the literature and proportional meta-analysis to assess prevalence and incidence of delirium in older adults with COVID-19, addressing differences according to sex, frailty status, and settings.

**Methods:**

We searched databases for English-language articles on prevalence and incidence of delirium in older adults with COVID-19, published between March 2020 and January 2023.

**Results:**

Of the 1171 articles identified, 66 met selection criteria and were included in the meta-analysis (*n *= 35,035 participants, age-range 66–90 years old, 46.6% females). We observed similar pooled prevalence (20.6% [95% Confidence Interval (CI) 17.8–23.8%]) and incidence (21.3% [95% CI 14.7–30%]) of delirium. Pooled occurrence (both prevalence and incidence) of delirium was similar according to sex (females 21.3% [95% CI 16–27.5%] vs. males 23.8%% [95% CI 18.2–30.4%], *p*-value = 0.55) and study setting (nursing homes 22.5% [95% CI 14.2–33.6%] vs. hospital 20.3% [95% CI 17–24%], *p* = 0.68), but it was significantly higher in frail versus non-frail patients (37% [95% CI 26.6–48.8%] vs. 12.5% [95% CI 7.8–19.6%], *p*-value < 0.01). Delirium definitions and assessment tools largely varied across studies.

**Conclusion:**

This review delineates delirium as a common feature of COVID-19, particularly in frail older adults, and supports its formal inclusion among COVID-19 symptoms. The considerable heterogeneity in delirium assessment highlights the need for an operational strategy to standardize definitions and tools utilization in the management of frail older adults.

**Supplementary Information:**

The online version contains supplementary material available at 10.1007/s41999-023-00906-7.

## Key summary points

**Aim:**

To conduct an updated systematic review of the literature and proportional meta-analysis to assess prevalence and incidence of delirium in older adults with COVID-19.

**Findings:**

Delirium is a prevalent feature of COVID-19 in older adults, especially those living with frailty. Delirium may be the only sign or symptom of COVID-19 in this population.

**Message:**

Delirium is common in older adults with COVID-19 and a formal inclusion as a COVID-19 feature is advisable.

**Supplementary Information:**

The online version contains supplementary material available at 10.1007/s41999-023-00906-7.

## Background

The coronavirus disease 2019 (COVID-19) pandemic, caused by severe acute respiratory syndrome coronavirus 2 (SARS-CoV-2), has triggered a global health crisis with a disproportionate impact on the older population. Multiple long-term chronic conditions, together with frailty, have been identified as relevant elements that contributed to an increased vulnerability of older adults to negative outcomes associated with SARS-CoV-2 infection, such as increased hospitalization and mortality rate [1, 2]. The elevated fatality rate observed among older adults, coupled with the increased demands of medical attention and care, has placed significant pressure on healthcare systems worldwide [3, 4]. As a result, there has been an urge since the beginning of the pandemic to develop effective ways to early detect infected individuals to reduce viral spread and outbreaks. Older adults with acute illness often present atypical symptoms and can manifest geriatric syndromes, such as delirium or falls, posing clinical challenges to an optimal management of older patients [5–7].

Delirium, an acute global brain dysfunction that usually occurs in the setting of physical illness [6], has been recognized as a presenting feature of COVID-19 and found to independently increase post-discharge mortality in older adults [8, 9]. Several studies have shown that a significant proportion of older people, especially those who are frail, can present with delirium as the only sign or symptom of SARS-CoV-2 infection [10]. This atypical onset of COVID-19 has been observed not only among hospitalized and institutionalized older individuals but also among community-dwellers [8, 11]. Furthermore, the rate of delirium in COVID-19 people admitted to the emergency department (ED) has been reported to be significantly higher in comparison to studies conducted in the same setting before the COVID-19 pandemic, even when considering similar risk factors [12]. Early recognition of delirium is essential to reduce adverse outcomes such as mortality, intensive care units (ICU) admissions, the need for ventilation, and increased length of stay of older adults hospitalized for COVID-19 [12–15]. A recent study showed that patients with delirium were four times more likely to die than those without delirium [14]. Similarly, a meta-analysis of nine studies showed that the death rate in COVID-19 patients with delirium was twice as high as those without delirium [13–17]. In this scenario, the unique social factors of an unprecedented pandemic, such as self-isolation and limited family visits, may have negatively impacted on the management of delirium [10]. A study has shown that visitation restriction increased delirium incidence in emergency inpatients regardless of the acute cause of admission, and that the absence of close family members can lead to delays in diagnosis and difficulties in implementing non-pharmacological treatments, further adding to the challenges in managing patients affected by delirium [18].

From the beginning of the pandemic, several meta-analyses and reviews have been published on the association between delirium and COVID-19. However, there has been a lack of definitive prevalence data across different settings, with some reviews mixing incidence and prevalence or failing to contextualize with frailty status [19].

## Objectives

The aim of the present study is to systematically review the literature and provide an updated pooled prevalence and incidence of delirium in older adults with COVID-19, addressing differences between males and females, frailty status, and study settings, shedding light on the unique aspects of delirium in this vulnerable population.

## Methods

We reviewed all studies providing information on the occurrence (prevalence and incidence) of delirium in older adults (> 65 years old or older) affected by SARS-CoV-2 infection, including those who were hospitalized, institutionalized, or were community-dwelling, regardless of study design, definition of delirium or type of assessment tool for delirium.

The protocol of the present study was registered a priori in the International prospective register of systematic reviews PROSPERO (registration number CRD42022366613) and was reported following the Preferred Reporting Items for Systematic Reviews and Meta-Analyses (PRISMA) recommendations. The PRISMA 2020 checklist is available in the Appendix. For the present study, no ethics committee approval was required.

### Search strategy and studies sources

We conducted a systematic review of studies retrieved from PubMed, Web of Science, and Google Scholar. We restricted the search to studies published in the English language between March 2020 and January 2023. Complete search strategies (available in the Appendix) were evaluated and optimized by an expert bibliographer prior to the commencement of the systematic review.

### Studies eligibility criteria, selection, and data extraction

The titles and abstracts of the studies were independently screened by two assessors (M.B.Z. and A.M.O.). Upon title screening, we identified the metanalysis by Shao et al. [19]. To ensure an updated search, we performed a hand-search of the reference lists of this metanalysis. Furthermore, the reference lists of the included articles were also hand-searched to identify any additional relevant studies.

Studies that reported the occurrence of delirium in older adults aged 65 years and older affected by SARS-CoV-2 infection and were either hospitalized or institutionalized with SARS-CoV-2 infection were selected. Delirium was classified as prevalent when it was identified as a presenting symptom of COVID-19, either at the onset of infection or on hospital admission. Delirium diagnosed during hospitalization was considered as incident delirium. Articles were excluded if they did not align with the aims of the review or did not present original data. Articles that included individuals younger than 65 years were still considered if the mean/median age of the study population was higher than 65 years old or if data were provided according to age groups, allowing for the extraction of subgroup data specifically for adults aged 65 years and older. Given our specific focus on older individuals with COVID-19, we selected studies that either included COVID-19 patients identified based on positive nasopharyngeal swab samples or by imaging findings suggestive of COVID-19-related pneumonia and provided non-aggregated data, that allowed for subgroup identification of the study population (i.e., nursing home residents versus staff members both infected by SARS-CoV2 infection) and subgroup data extraction, when necessary. When a study presented data on two different populations, both eligible for the scope of this review (e.g., community-dwellers group versus hospitalized group), we considered them as separate study groups, assigning them a unique identifier based on the first author’s name followed by an ordinal number. Delirium definitions were screened in each included article and information on definition of delirium or diagnostic instrument used to support the diagnosis were recorded. Similarly, we screened each included study for the use of a delirium assessment tool and evaluation of frailty status. We finally registered the type of tool used to assess frailty as well as the study setting categorized into “Hospital”, “Nursing Home”, and “Community” accordingly to where the study took place. Long-term care units, nursing homes, and assisted-living facilities were all classified as “Nursing Home”. For each article, the following data were collected: number of COVID-19 patients, number of COVID-19 patients experiencing delirium, mean/median age and sex distribution (number of females, %) of the study population, number of females and males diagnosed with delirium, frailty status, mean/median length of hospital stay of the study population and number of deaths among patients with delirium.

The full text of the articles selected by at least one of the assessors underwent further evaluation. The same assessors independently extracted information from the selected studies. Any disagreement was resolved through consensus.

### Assessment of risk of bias

The quality of the included studies was evaluated independently by the two assessors through the Newcastle Ottawa Scale (NOS), a tool for the qualitative evaluation of observational studies. For cross-sectional studies, the assessors used a modified version of the NOS specifically designed for such study designs [20]. The decision was made based on the work by Moskalewicz et al. [21], which found no significant difference between the use of the modified-NOS scale and the Appraisal Tool for Cross-Sectional Studies [22]. The choice was based on the simplicity of the tool and time optimization.

Studies with scores of > 7 indicated low risk of bias, scores of 4–7 indicated moderate risk of bias, and scores of < 4 indicated high risk of bias. Any disagreement in quality assessment was resolved through consensus. The likelihood of publication bias was assessed via funnel plots (available in the Appendix) and Egger’s test.

### Data analysis

For each measure of interest (prevalence and incidence), we ran a proportional meta-analysis. Considering the observational design of the retrieved studies and the methodological differences between them, pooled proportions were obtained through random effect models and Mantel–Haenszel weighting. The lack of homogeneity within the pooled studies was assessed through the *I*^2^ statistics (significant if ≥ 50%), which provides an estimate of the proportion of variability explained by differences between the included studies. Analyses are presented according to the prevalence and incidence of delirium. For the subgroup analysis by sex, frailty status and study settings, we consider the occurrence of delirium (both prevalence and incidence) as a single measure of interest. Results are presented as forest plots. Publication bias was assessed by mean of Egger’s test and funnel plot. All statistical analyses were performed with R Studio (R Core Team Version 4.2.1) [23] using the “meta” and “metafor” packages [24, 25].

## Results

**Fig. 1:**
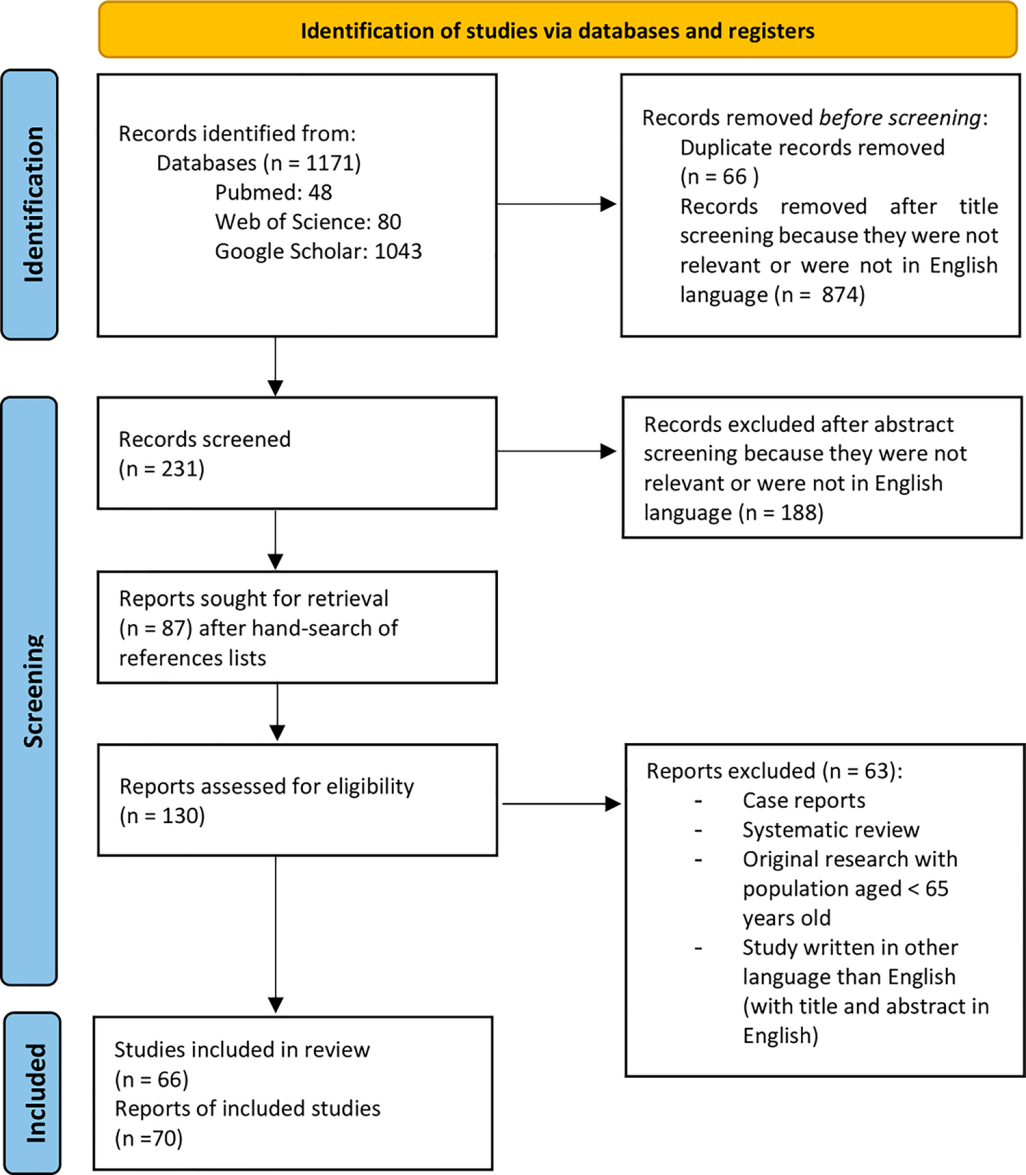
PRISMA 2020 flow diagram

Through the literature search, we retrieved 1171 articles. After excluding duplicates and articles that were not written in English, we screened 231 works and hand-searched the references lists. We finally assessed for eligibility of 130 articles for full-text reading. We excluded 63 of the screened full text due to the study design (case reports and reviews), inclusion of participants younger than 65 years old, and/or the mean/median age of the study population was less than 65 years. Additionally, we excluded 2 articles that were not written in English but had titles and abstracts in English, leading to a final number of 66 articles included in the present review. PRISMA 2020 flow diagram of the identification of eligible articles is presented in Fig. 1.

Main characteristics and findings of selected studies are summarized in Supplementary Table S1a and S1b of the Appendix. The overall number of participants was 35,035, with a mean/median age ranging from 66 to 90 years old; among the participants 46.6% were females, with a prevalence ranging from 22 to 78% (Supplementary Table S1b).

Most of the studies were carried out in Europe between Italy (*n* = 16, including one study co-conducted with Spain) and the United Kingdom (*n* = 16, including one study with an international multicenter designed. Remaining studies in European countries were conducted in: France (*n* = 6), Spain (= 5, including one study co-conducted with Italy), Netherlands (= 4), Belgium (*n* = 2), Sweden (*n* = 1), Germany (*n* = 1), Poland (*n* = 1), Norway (*n* = 1) and Switzerland (*n* = 1). A total of 9 studies were conducted in North and South America (USA = 4, Canada = 3, Brazil = 2). The rest of the studies were conducted in India (*n* = 1), Iran (*n* = 1), China (*n* = 1), Japan (*n* = 1) and Turkey (*n* = 1).

Most studies (58/66) enrolled patients admitted to acute hospital or emergency department. None of the studies specifically focused on community-dwellers, but two studies compared this population to nursing home and acute hospital populations, respectively. Additionally, nine studies were carried out in long-term care facilities or nursing homes, and one study was conducted in assisted-living facilities (see Supplementary Table S1a for referral).

Out of the total sixty-six studies included in the analysis, 50% (*n* = 33) reported a specific definition of delirium and/or clearly stated that a clinical assessment was conducted by a trained physician or nurse. Among these studies, in four of them, the clinical evaluation was performed by a psychiatrist, while in most of the remaining studies, patients were evaluated either by clinicians on the day of admission or by geriatricians. Among the thirty-three studies that provided a delirium definition, sixteen of them explicitly referred to the standard delirium assessment based on the Diagnostic and Statistical Manual of Mental Disorders, Fifth Edition (DSM-5), as the defining criteria for delirium. In other studies, delirium was classified under broader terms such as "confusion" or "altered mental status." Notably, 75% (*n* = 25) of the studies that reported a definition of delirium also mentioned the concomitant use of a delirium assessment tool other than the DSM-5. Only five studies relied solely on the DSM-5 for delirium assessment. We identified the use of 13 different tools, including the DSM. The most commonly used tools were the “Confusion Assessment Method (CAM)” and “Assessment test for delirium & cognitive impairment (4AT)”. In one study, it was clarified that comprehensive geriatric assessment (CGA) was performed on all admitted patients. All delirium definitions and used assessment tools are summarized in Supplementary Table S1a.

Twenty-seven studies (41%) assessed frailty, with the most used assessment tool being the Clinical Frailty Scale (CFS). One study calculated a Frailty Index, another used the Frailty Phenotype Criteria. The other two used tools were represented by the Frail Non-Disabled survey (FIND), and the Program on Research for Integrating Services for the Maintenance of Autonomy-7 (PRISMA-7).

**Fig. 2:**
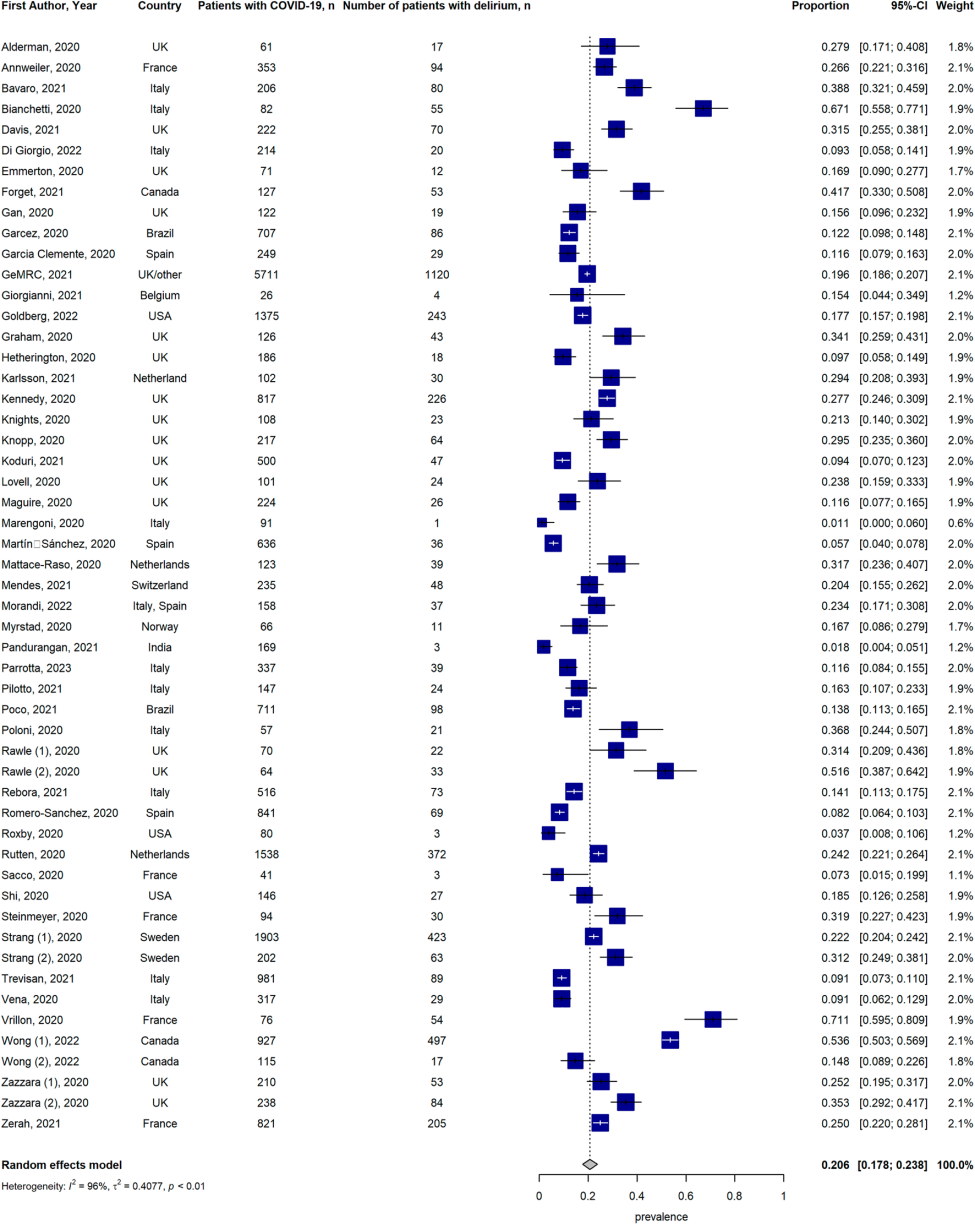
Forest plot of the pooled prevalence of delirium in older adults affected by COVID-19

**Fig. 3:**
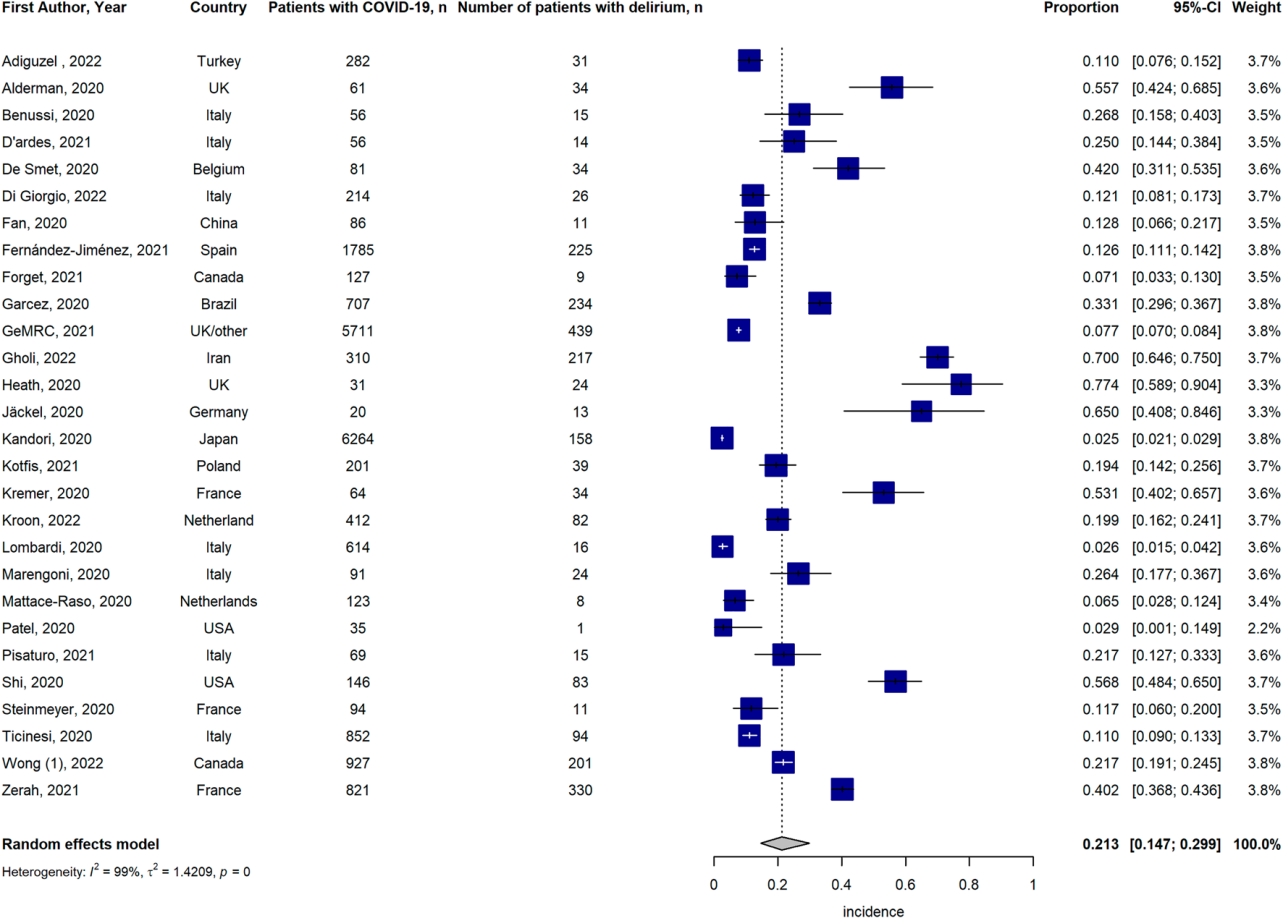
Forest plot of the pooled incidence of delirium in older adults affected by COVID-19

A total of 49 studies reported data on the prevalence of delirium in older adults affected by COVID-19, 28 studies reported data on the incidence of delirium and 11 studies reported information on both. The forest plot of the pooled proportion of delirium occurrence is presented in Fig. 2, showing a delirium prevalence of 20.6% [95% Confidence Interval (CI) 17.8–23.8%] while the forest plot of the pooled proportion of incidence of delirium is presented in Fig. 3, indicating an incidence of 21.3% [95% CI 14.7–30%]. A high level of heterogeneity *I*^2^ was present in both proportional meta-analyses. The Egger’s test, performed to assess publication bias, yielded non-significant *p*-values for both prevalence and incidence data (respectively, *p*-value = 0.54 and *p*-value = 0.23). However, the visual methods of checking for asymmetry via funnel plots show potential bias, especially in studies reporting the incidence of delirium (Supplementary Fig. S1a, b). Finally, overall occurrence (both prevalence and incidence) of delirium was 21.0% [95% CI 17.8–24.3%] (Supplementary Fig. S2). For the subgroup analysis based on sex, we consider 18 studies that reported the occurrence of delirium (prevalence and incidence) in both males and females. The pooled occurrence of delirium according to sex is presented in Supplementary Fig. S3 and show a non-significant difference between females and males (21.3% [95% CI 16–27.5%] vs. 23.8% [95% CI 18.2–30.4%], *p*-value = 0.55). A similar analysis was performed for frailty status. Data on the occurrence of delirium according to frailty status were available in 7 studies, presenting 8 study populations (Supplementary Fig. S4), showing a significant difference in the occurrence between frail and non-frail participants (37.0% [95% CI 26.6–48.8%] vs. 12.5% [95% CI 7.8–19.6%] respectively, *p*-value < 0.01). Furthermore, we performed a sub-analysis according to the study setting. Due to the limited number of studies reporting data on community-dwelling older adults affected by COVID-19—with a total sample size of 308 observations and a total number of events of 108—we did not perform a metanalysis for this setting. Supplementary Fig. S5 shows data on the occurrence of delirium accounting to the study setting. A slightly higher occurrence of delirium was reported in nursing home settings (22.5% [95% CI 14.2–33.6%]) compared to hospital settings (20.3% [95% CI 17–24%], *p* = 0.68), although the difference was not significant.

## Discussion

We conducted an updated systematic review on the occurrence of delirium in COVID-19 patients, specifically focusing on adults aged 65 years and older, and our analysis included a total of 66 studies. The proportional meta-analysis showed that almost one-fifth of older patients with COVID-19 presented delirium during the course of the infection, delineating delirium as a frequent symptom of COVID-19.

Though several articles have been published since the beginning of the pandemic, identifying delirium as a presenting symptom of COVID-19, it is important to note that, at the time of writing, neither delirium nor confusion are listed as official feature of COVID-19 by major governmental institutions. Only the website of the World Health Organization (WHO) does include confusion as one of the serious symptoms of COVID-19. After almost three years of dealing with this novel type of Coronavirus, it seems detrimental that a formal inclusion of delirium as a COVID-19 sign/symptom should take place. Furthermore, while delirium is more commonly observed in older adults and individuals with acute illness [6], there have been reports of delirium in younger patients affected by severe COVID-19, particularly those requiring intensive care unit (ICU) admission or mechanical ventilation [26]. This could potentially explain the difference in delirium incidence between our meta-analysis and the meta-analysis conducted by Shao et al. [19], which also included studies with younger patients. An older target population, which may have other predisposing factors of delirium, such as cognitive impairment and frailty [6], could potentially exhibit a similar proportion of both delirium prevalence and incidence, as older adults are intrinsically more prone to experience geriatric symptoms even in the case of mild illness. This vulnerability may allow delirium to manifest as the only feature of COVID-19. On the contrary, younger patients might experience delirium predominantly in case of severe distress, thus explaining the increased incidence of delirium during the acute phase, potentially undetected by our metanalysis that is exclusively focusing on the older population.

Second, we found that delirium occurred more frequently in male and frail patients. A higher occurrence of delirium in male patients might be related to the role of sex in predicting severe COVID-19 illness. Male sex, obesity, and multiple comorbidities are clinical features that have been found to be associated with negative outcomes, including the development of acute respiratory distress syndrome (ARDS), multi-organ failure syndrome (MOFS), and ultimately, mortality [27, 28]. Additionally, there is a possibility that delirium is more common among male individuals due to an increased severity of COVID-19. Frailty is associated with adverse outcomes in COVID-19 patients, increasing the risk of related mortality and care needs in survivors [29]. Frailty and delirium are intrinsically related and represent multifactorial conditions that share many common pathophysiological aspects, such as the potential involvement of inflammatory pathways, and both have implications for negative health-related outcome [30]. Although the data on the occurrence of delirium in frail patients were assessed in a relatively small number of studies, the significant difference in the delirium occurrence between frail and non-frail patients underlines the importance of frailty as a contributing factor in the onset of delirium in COVID-19 patients. This should be taken in consideration when addressing the older population. Furthermore, we noted that the prevalence of delirium varied depending on the study setting. We found a moderately higher prevalence of delirium in the nursing home population, which may reflect the higher level of frailty among those already institutionalized. In long-term care settings, especially during the first stage of the pandemic, restrictive measures were set in place to avoid outbreaks and viral spread among residents and staff member [3]. Facilities were forced to increase social isolation and decrease family visitations, thus reducing the possibilities to resort to conventional non-pharmacological methods to prevent delirium—such as re-orientation and social interaction, early mobilization, pain management, avoidance of physical restraints—other than just pharmacological solutions [3, 10].

Finally, while frailty was evaluated using the same tool (CFS) in most studies, the definitions of delirium were heterogeneous across different studies and there was a high variability in the choice of the delirium assessment tool. Only 16 studies explicitly referred to the standard delirium assessment based on DSM-V criteria. Amongst the studies that reported using a standardized assessment tool, the CAM and the 4AT tools were the most commonly used. A multicenter prospective diagnostic study published in 2019 by Shenkin et al. performed a sensibility and sensitivity analysis to compare the accuracy of CAM and 4AT to detect delirium during hospitalization and showed a higher sensitivity and slightly lower specificity of the 4AT compared to the CAM tool. As highlighted by the authors, evidence suggests that the 4AT is a valid and rapid tool comparable to the CAM when used by a trained assessor. However, the performance of the CAM may be compromised if the assessor is not familiar with the tool [31]. Overall, in clinical daily practice, both tools are easy and rapid to use for physicians treating this population. At the same time, it is possible that the working conditions of healthcare professionals during the pandemic, along with the use of personal protective equipment that made the administration of tools relying on verbal response difficult, may have discouraged the adoption of delirium detection tools. Nevertheless, it has been shown that, without using specific instruments, the ability to detect delirium is very poor [32, 33]. Despite O’Hanlon and colleagues defining delirium as “a missing piece in the COVID-19 pandemic puzzle” back in May 2020 [34], there has been little systematic effort to enhance the prevention and management of delirium in COVID-19 patients, as restrictive measures and self-isolations remain cardinal indications in the prevention of SARS-CoV-2 infection. The recognition of delirium as a key feature of COVID-19 by governmental institutions might help the implementation of delirium detection tools thereby reducing the risk of under-detecting SARS-CoV2 infection, especially when delirium represents the sole presenting feature.

### Strength and limitations

To conduct this study, we used a comprehensive search strategy and systematic review method, consulting several databases in the process, allowing clinically relevant conclusions in terms of overall delirium rates in COVID-19 patients aged 65 years and older. However, several limitations should be considered. First, the studies included in this analysis exhibited heterogeneity in terms of their study samples and methods for collecting information on outcome variables. In particular, none of the included studies specifically focused on adults with intellectual disabilities or older adults affected by psychiatric conditions, special populations intrinsically at higher risk of delirium in the case of an acute illness and most studies assessed delirium using a dichotomous approach that may have biased the study results. Additionally, the lack of information regarding whether assessors were blinded to the outcome represent a potential source of bias. Second, the variability in the assessment tool used to diagnose delirium among the included studies may have led to the under-recognition of certain cases of delirium in COVID-19 patients, especially since types of delirium—hyperactive, hypoactive or mixed were rarely assessed. Third, not all included studies specifically focused on investigating delirium in older COVID-19 patients and/or investigated delirium only upon hospital admission, leading to missing data for several variables of interest, especially incidence. Fourth, most studies were conducted in hospital settings, with only a few studies providing data from long-term care facilities or community settings, thus allowing only a pooled prevalence calculation. Lastly, a possible publication bias cannot be excluded, although it did not seem to have influenced the measures of proportion substantially.

## Conclusion

We conducted an updated systematic review and meta-analysis involving 66 studies and a total of 35,035 COVID-19 older patients. We found similar prevalence and incidence of delirium in COVID-19 older patients, delineating delirium as an important symptom of COVID-19, especially in frail older adults. Formal inclusion of delirium as a COVID-19 feature in older adults is advisable. The high heterogeneity in the assessment of delirium highlights the need for an operational strategy to standardize definitions and tools utilization to facilitate the integration of delirium assessment in daily clinical practice, especially when treating older and frail population. Our findings underline the need for healthcare professionals to consider the impact of delirium in this population to optimize strategies to prevent and manage delirium, especially in the context of a pandemic.

### Supplementary Information

Below is the link to the electronic supplementary material.

Supplementary file 1 (DOCX 1305 KB)

## Data Availability

Data is available upon written request to the corresponding author.
