# Supplementary material for: A pandemic of delirium: an updated systematic review and meta-analysis of occurrence of delirium in older adults with COVID-19
Source: Eur Geriatr Med. 2024 Mar 18;15(2):397–406. doi: 10.1007/s41999-023-00906-7 (PMC10997697; doi:10.1007/s41999-023-00906-7)
Supplement: Supplementary file 1 — (DOCX 1305 KB) [file 41999_2023_906_MOESM1_ESM.docx]

**A pandemic of delirium: an updated systematic review and metanalysis of occurrence of delirium in older adults with COVID-19**

**APPENDIX**

- **Appendix 1**
  - **Search strategy**
- **Appendix 2**
  - **Supplementary Table 1a**
  - **Supplementary Table 1b**
  - **Supplementary Figure 1a**
  - **Supplementary Figure 1b**
  - **Supplementary Figure 2**
  - **Supplementary Figure 3**
  - **Supplementary Figure 4**
- **Appendix 3**
  - **Prisma Check List**
- **Appendix 4**
  - **Reference List of Included Articles**

**Appendix 1**

***Search strategy***

**PUBMED**

("Prevalence"[MeSH Terms] OR ("prevalence*"[Title/Abstract] OR "period prevalence*"[Title/Abstract] OR "point prevalence*"[Title/Abstract])) AND ("Delirium"[Title/Abstract] OR "Confusion"[Title/Abstract] OR "confusional state*"[Title/Abstract]OR "Disorientation"[Title/Abstract] OR "Bewilderment"[Title/Abstract] OR "neurologic* manifestation*"[Title/Abstract] OR "neurologic* deficit*"[Title/Abstract] OR "agitation"[Title/Abstract] OR "Confusion"[MeSH Terms] OR "Neurologic Manifestations"[MeSH Terms]) AND ("Aged"[MeSH Terms] OR "Frail Elderly"[MeSH Terms] OR ("frail elder*"[Title/Abstract] OR "frail older adult*"[Title/Abstract] OR "older adult*"[Title/Abstract] OR"elderly"[Title/Abstract] OR "Aged"[Title/Abstract])) AND ("Hospitalization"[MeSH Terms] OR "Nursing Homes"[MeSH Terms] OR ("hospitalization*"[Title/Abstract] OR "nursing home*"[Title/Abstract])) AND ("covid 19"[MeSH Terms] OR "sars cov 2"[MeSH Terms] OR ("covid 19"[Title/Abstract] OR "covid 19"[Title/Abstract] OR "sars cov 2"[Title/Abstract] OR "sars cov2"[Title/Abstract] OR "coronavirus"[Title/Abstract] OR "2019 ncov"[Title/Abstract] OR "2019 ncov"[Title/Abstract]))

**WEB OF SCIENCE**

Search restricted to “topic”:

(“delirium” OR “confusion” OR “agitation” OR “neurologic* manifestation*”) AND ("COVID-19" OR "coronavirus" OR "Sars-CoV-2" OR " SARS-CoV-2" OR "corona*" OR " sars-cov-2" OR "Severe Acute Respiratory Syndrome Coronavirus 2 multimorbid*")AND (“Aged” OR "Frail Elderly" OR "frail elder*" OR "frail older adult*" OR "older adult*" OR"elderly") AND ("Hospitalization*" OR "Nursing Home*")

**GOOGLE SCHOLAR**

Search restricted to scientific articles

Prevalence + (delirium OR confusion OR "neurologic* manifestation*" OR agitation) + ("COVID-19”OR corona* OR "Sars-CoV-2”) + ("old* adults” OR "elderly” OR "frail adults") + (Hospitalization* OR“Nursing Home*"

**Appendix 2**

**Supplementary Table 1a**: Characteristics of the studies included in the systematic review (continues in Table 1b)

| **First author, Year,  Contry** | **Design of**  **the study  and  study name** | **Study Setting** | **Explicit delirium definition present (YES/NO) †** | **Type of definition** | **Use of assessment tool (YES/NO)** | **Delirium  assessment  tool** | **Assessessment  of frailty status** | **Frailty  assessment  tool** | **NOS score** | **Risk of bias** |
| --- | --- | --- | --- | --- | --- | --- | --- | --- | --- | --- |
| Adiguzel^1^, 2022 Turkey | Retrospective | Hospital | YES | presence of delirium per  clinical assessment  (psychiatric evaluation) | YES | DSM-5^(1)^ | NA | NA | 4 | Moderate |
| Alderman^2^, 2022 UK | Prospective | Palliative Care Hospital | YES | Presence of delirium  per clinical assessment | YES | RASS^(2)^ | NA | NA | 3 | High |
| Annweiler^3^, 2021 France | Cross-sectional,  National French Survey of Coronavirus Disease | Hospital,  Nursing home | YES | Presence of delirium  per clinical assessment | NO | NA | NA | NA | 3 | High |
| Bavaro^4^,  2021 Italy | Prospective case-series | Hospital | YES | As mental  confusion | NO | NA | YES | CFS* | 5 | Moderate |
| Benussi^a,5^, 2020 Italy | Retrospective single centre | Hospital | YES | Presence of delirium  per clinical assessment | YES | CAM^(3)^ | NA | NA | 7 | Moderate |
| Bianchetti^6^, 2020 Italy | Retrospective | Hospital | YES | Presence of delirium  per clinical assessment | NO | NA | NA | NA | 3 | High |
| D'ardes^7^,  2021 Italy | Prospective | Hospital | YES | Presence of delirium  per clinical assessment | YES | 4AT^(4)^, CAM-ICU^(5)^ | NA | NA | 7 | Moderate |
| Davis^8^,  2021 UK | Retrospective | Hospital | YES | Presence of delirium  per clinical assessment | YES | 4AT^(4)^ | YES | CFS* | 5 | Moderate |
| De Smet^9^, 2020 Belgium | Retrospective | Hospital | NO | Presence of delirium  per clinical assessment | NO | NA | YES | CFS* | 6 | Moderate |
| Di Giorgio^10^, 2022 Italy | Retrospective | Hospital | YES | DSM-5, presence of delirium  per clinical assessment  (psychiatric evaluation) | YES | DSM-5^(1)^, 4AT^(4)^ | NA | NA | 8 | Low |
| Emmerton^11^, 2020 UK | Retrospective | Hospital | NO | NA | NO | NA | NA | NA | 2 | High |
| Fan^12^,  2020 China | Retrospective | Hospital | YES | presence of delirium | YES | DSM-6^(1)^ | NA | NA | 4 | Moderate |
| Fernández-Jiménez^13^, 2021 Spain | Prospective | Hospital | YES | presence of delirium  per clinical assessment  (psychiatric evaluation) | YES | DSM-5^(1)^ | NA | NA | 8 | Low |
| Forget^14^,  2021 Canada | Retrospective | Hospital | YES | DSM-5,  presence of delirium | YES | DSM-5^(1)^, Validated  tool^(6)^ | NA | NA | 7 | Moderate |
| Gan^15^,  2020 UK | Retrospective | Hospital | NO | As confusion | NO | NA | YES | CFS* | 5 | Moderate |
| Garcez^16^,  2020 Brazil | Retrospective cohort,  CO-FRAIL study | Hospital | YES | Presence of delirium  per clinical assessment | YES | CHART-DEL^(7)^ | NA | NA | 8 | Low |
| Garcia Clemente^17^, 2020 Spain | Prospective cohort | Hospital | NO | As confusion | NO | NA | NA | NA | 5 | Moderate |
| GeMRC^18^, 2021 UK/other | Retrospective cohort,  Covid Collaborative | Hospital | YES | Presence of delirium  per clinical assessment | YES | 4AT^(4)^ | YES | CFS* | 8 | Low |
| Gholi^19^,  2022 Iran | Prospective | Hospital | YES | Presence of delirium  per clinical assessment | YES | CAM-ICU^(5)^ | NA | NA | 7 | Moderate |
| Giorgianni^20^, 2021 Belgium | Case-series | Hospital | NO | As confusion | NO | NA | NA | NA | 3 | High |
| Goldberg^b,21^, 2022 USA | Retrospective cohort, RECOVER Network registry | Hospital | NO | As altered mental status | NO | NA | NA | NA | 7 | Moderate |
| Graham^c,22^, 2020 UK | Cross-sectional | Nursing home | NO | As confusion/ altered behaviour | NO | NA | NA | NA | 3 | High |
| Heath^23^,  2020 UK | Survey | Palliative Care Hospital | NO | Presence of delirium  per clinical assessment | NO | NA | NA | NA | 2 | High |
| Hetherington^24^,  2020 UK | Retrospective cohort | Palliative Care Hospital | NO | Presence of delirium  per clinical assessment | NO | NA | NA | NA | 3 | High |
| Jäckel^a,25^, 2020 Germany | Cross-sectional | Hospital | YES | Presence of delirium  per clinical assessment | YES | NuDesc^(8)^,  RASS^(2)^ | NA | NA | 2 | High |
| Kandori^26^, 2020 Japan | Retrospective cohort | Hospital | YES | presence of delirium  per clinical assessment  (psychiatric evaluation) | YES | DSM-5^(1)^ | NA | NA | 7 | Moderate |
| Karlsson^27^, 2021  Netherland | Retrospectivecohort | Hospital | NO | As confusion | NO | NA | NA | NA | 4 | Moderate |
| Kennedy^28^, 2020 UK | Multicentric retrospective  cohort | Hospital | YES | Presence of delirium  per clinical assessment, delirium characteristics listed | YES | CAM^(3)^ | NA | NA | 7 | Moderate |
| Knights^29^, 2020 UK | Retrospective cohort | Hospital | NO | Presence of delirium  per clinical assessment | NO | NA | YES | CFS* | 3 | High |
| Knopp^30^,  2020 UK | Prospective cohort | Hospital | YES | Presence of delirium  per clinical assessment | NO | NA | YES | CFS* | 3 | High |
| Koduri^31^,  2021 UK | Cross-sectional | Hospital | NO | As confusion | NO | NA | YES | CFS* | 5 | Moderate |
| Kotfis^32^,  2021 Poland | Retrospective cohort | Hospital | YES | Presence of delirium  per clinical assessment | YES | DSM-5^(1)^ | YES | CFS* | 8 | Low |
| Kremer^33^, 2020 France | Retrospective | Hospital | NO | As confusion | NO | NA | NA | NA | 2 | High |
| Kroon^34^,  2022 Netherland | Multicenter cohort,  COVID‐OLD | Hospital | YES | DSM-5,  presence of delirium  per clinical assessment | YES | DSM-5^(1)^,  DOSS^(9)^ | YES | CFS* | 7 | Moderate |
| Lombardi^35^, 2020 Italy | Cross-sectional | Hospital | NO | Presence of delirium  per clinical assessment | NO | NA | NA | NA | 5 | Moderate |
| Lovell^36^,  2020 UK | Retrospective | Hospital | NO | Presence of delirium  per clinical assessment | NO | NA | NA | NA | 2 | High |
| Maguire^37^, 2020 UK | Cross-sectional | Hospital | NO | Presence of delirium  per clinical assessment | YES | NEWS^(10)^ | YES | CFS* | 6 | Moderate |
| Marengoni^38^, 2020 Italy | Retrospective | Hospital | YES | presence of delirium  per clinical assessment | YES | DSM-5^(1)^, 4AT^(4)^ | YES | CFS* | 8 | Low |
| Martín‑  Sánchez^b39^,  2020 Spain | Retrospective cohort,  COVID-19_URG-HCSC registry | Hospital | NO | As confusion | NO | NA | NA | NA | 5 | Moderate |
| Mattace-Raso^40^,  2020 Netherlands | Cross-sectional | Hospital | YES | DSM-5,  presence of delirium  per clinical assessment | YES | DSM-5^(1)^, DOSS^(9)^,  ICDSC^(11)^ | NA | NA | 2 | High |
| Mendes^41^, 2021  Switzerland | Retrospective | Hospital | YES | DSM-5,  presence of delirium  per clinical assessment | YES | DSM-5^(1)^, CAM^(3)^ | YES | CFS* | 8 | Low |
| Morandi^42^, 2022 Italy, Spain | Prospective cohort | Hospital | YES | Presence of delirium  per clinical assessment | YES | 4AT^(4)^ | YES | CFS* | 7 | Moderate |
| Myrstad^43^, 2020 Norway | Prospective cohort | Hospital | NO | As confusion | YES | NEWS2^(12)^ | YES | CFS* | 3 | High |
| Pandurangan^44^  2021 India | Retrospective | Hospital | NO | As other CSN  symptoms | NO | NA | NA | NA | 2 | High |
| Parrotta^45^, 2023 Italy | Retrospective cohort,  GeroCovid Observational Study | Hospital | YES | Presence of delirium  per clinical assessment | YES | CGA^(13)^ | YES | Fried Criteria** | 6 | Moderate |
| Patel^c,46^,  2020 USA | Prospective | Nursing home | NO | As loss of consciousness  or delirium | NO | NA | NA | NA | 2 | High |
| Pilotto^a,47^, 2021 Italy | Prospective | Hospital | NO | As Delirium/ Altered Mental State | NO | NA | NA | NA | 3 | High |
| Pisaturo^d,48^, 2021 Italy | Case-control | Hospital | NO | Presence of delirium  per clinical assessment | NO | NA | NA | NA | 2 | High |
| Poco^49^,  2021 Brazil | Longitudinal observational study,  CO-FRAIL Project | Hospital | NO | As acute  confusion | YES | CHART-DEL^(7)^ | YES | CFS* | 8 | Low |
| Poloni^50^,  2020 Italy | Retrospective single centre | Long-term care | YES | DSM-5,  presence of delirium  per clinical assessment | YES | DSM-5^11)^, CAM^(3)^ | NA | NA | 7 | Moderate |
| Rawle (1)^51^, 2020 UK | Retrospective cohort | Community | YES | DSM-5,  presence of delirium  per clinical assessment | YES | DSM-5^(1)^, 4AT^(4)^ | YES | CFS* | 7 | Moderate |
| Rawle (2)^51^, 2020 UK | Retrospective cohort | Nursing home | YES | DSM-5,  presence of delirium  per clinical assessment | YES | DSM-5^(1)^, 4AT^(4)^ | YES | CFS* | 7 | Moderate |
| Rebora^52^, 2021 Italy | Retrospective cohort | Hospital | YES | DSM-5,  presence of delirium  per clinical assessment | YES | DSM-5^(1)^, 4AT^(4)^, mRASS^(14)^ | NA | NA | 7 | Moderate |
| Romero-Sanchez^53^, 2020 Spain | Retrospective cohort,  ALBACOVID registry | Hospital | NO | As acute  confusional  syndrome | NO | NA | NA | NA | 5 | Moderate |
| Roxby^c,54^, 2020 USA | prospective | Assisting living facilities | NO | As confusion | NO | NA | NA | NA | 2 | High |
| Rutten^55^,  2020 Netherlands | Prospective cohort | Nursing home | YES | As delirium,  confusion or  drowsines | NO | NA | NA | NA | 5 | Moderate |
| Sacco^c,56^,  2020 France | Retrospective cohort | Nursing home | NO | Presence of delirium  per clinical assessment | NO | NA | NA | NA | 4 | Moderate |
| Shi^a,57^,  2020 USA | Retrospective cohort | Long-term care | YES | As change in mental status  or lethargy/ presence of delirium  per clinical assessment | NO | NA | YES | Frailty Index*** | 5 | Moderate |
| Steinmeyer^58^, 2020 France | Retrospective cohort | Hospital | YES | Presence of delirium  per clinical assessment | YES | CAM^(3)^ | YES | FIND**** | 5 | Moderate |
| Strang (1)^59^, 2020 Sweden | Cross-sectional,  Swedish Register of Palliative Care (SRPC) | Nursing home | NO | Presence of delirium  per clinical assessment | NO | NA | NA | NA | 3 | High |
| Strang (2)^59^, 2020 Sweden | Cross-sectional,  Swedish Register of Palliative Care (SRPC) | Hospital | NO | Presence of delirium  per clinical assessment | NO | NA | NA | NA | 3 | High |
| Ticinesi^60^,  2020  Italy | Retrospective | Hospital | YES | Presence of delirium  per clinical assessment | YES | CAM^(3)^ | NA | NA | 7 | Moderate |
| Trevisan^61^, 2022 Italy | Retrospective cohort,  GeroCovid Observational | Hospital | NO | Presence of delirium  per clinical assessment | NO | NA | NA | NA | 8 | Low |
| Vena^62^, 2020 Italy | Retrospective cohort,  GECOVID-19 | Hospital | YES | As mental  confusion | NO | NA | NA | NA | 5 | Moderate |
| Vrillon^63^, 2020 France | Prospective cohort | Hospital | YES | As confusion or  presence of delirium  per clinical assessment | NO | NA | YES | CFS* | 6 | Moderate |
| Wong (1)^64^, 2022 Canada | Retrospective cohort | Hospital | YES | Presence of delirium  per clinical assessment | YES | CAM^(3)^ | YES | CFS* | 7 | Moderate |
| Wong (2)^64^, 2022 Canada | Retrospective cohort | Long-term care, rehabilitation unit | YES | Presence of delirium  per clinical assessment | YES | CAM^(3)^ | YES | CFS* | 7 | Moderate |
| Zazzara (1)^65^, 2020 UK | Retrospective cohort,  COVIDCollab study and  COVID Symptom Study app | Hospital | YES | Presence of delirium  per clinical assessment | YES | 4AT^(4)^ | YES | CFS* | 8 | Low |
| Zazzara (2)^65^, 2020 UK | Retrospective cohort,  COVIDCollab study and  COVID Symptom Study app | Community | YES | Do you have any of the  following symptoms: confusion, disorientation or drowsiness? Yes/No | NO | NA | YES | PRISMA-7***** | 7 | Moderate |
| Zerah^66^,  2021 France | Multicentric retrospective cohort,  COVID19-APHP Group | Hospital | YES | Presence of delirium  per clinical assessment | YES | CAM^(3)^ | YES | CFS * | 8 | Low |
| NOTE TO INCLUSION CRITERIA  ^a^only subset of covid positive patients; ^b^only subgroup over 65; ^c^only subset of covid residents patients (not staff memeber); ^d^combined data of cases and controls (with and without dementia).  †YES was assigned in the case of a provided definition of delirium by the authors (in both tables and text) or whether it was specified that a clinical assessment was performed.  NO was assigned in cases where a clinical assessment of delirium was implied it is not specified (we can assume presence of delirium per clinical assessment)  Delirium as a presenting sintoms of COVID-19, at the onset of infection or on hospital admission was considered as prevalent.  Delirium diagnosed during hospitalization was considered as incident.  DELIRIUM ASSESSMENT TOOL   1. Diagnostic and Statistica Manual of mental disorder (DSM-V; DSM-VI) 2. Richmond Agitation and Sedation Scale score (RASS) 3. Confusion Assessment Method (CAM) 4. Assessment test for delirium & cognitive impairment (4AT) 5. Confusion Assessment Method – Intensive Care Unit (CAM – ICU) 6. Validate tool to retrospectively assess delirium by Kuhn et al. 2014;9 (11):e111823. 7. Chart-based Delirium Identification Instrument (CHART-DEL) 8. Nursing delirium screening scale (NuDesc) 9. Delirium Observation Screening Scale (DOSS) 10. National Early Warning Score(NEWS) 11. Intensive Care Delirium Screening Checklist (ICDSC) 12. National Early Warning Score2(NEWS2) 13. Comprehensive Geriatric Assessment (CGA) 14. Modified Richmond Agitation and Sedation Scale score (m-RASS)   FRAILTY ASSESSMENT TOOL  Roockwood Clinial Frailty Scale (CFS)*  Linda Fried Frailty Phenotypes Criteria **  Roockwood Frailty Index ***  Frail Non-Disabled survey (FIND) by Cesari et al. 2014 Jul 7;9(7):e101745.****  Program on Research for Integrating Services for the Maintenance of Autonomy- 7 (PRISMA-7) by Raîche et la. 2008 Jul-Aug;47(1):9-18.***** | | | | | | | | | | |

|  | |  |
| --- | --- | --- |
|  |  |  |

**Supplementary Table 1b**: Characteristics of the studies included in the systematic review (continues from Table 1a)

| **First author,**  **Year,**  **Contry** | **Age cut off for study inclusion in metanalysis^‡^** | **Patients with COVID-19, n** | **Age  [Mean (SD) - median (range)]** | **Females, %** | **Length of  hospital stay in days [Mean (SD) - median (range)]** | **Length of  hospital stay in days in patients with delirium  [Mean (SD) - median (range)]** | **Mortality in**  **patients with**  **delirium** | **Age of patients with delirium [Mean (SD) - median (range)]** |  |
| --- | --- | --- | --- | --- | --- | --- | --- | --- | --- |
| Adiguzel,  2022 Turkey | Study population included, age >65 y | 282 | 73.7(6.8) | 126(44.7%) | 10.9(11.3) | 12.3(12.5) | 15(48.4%) | 73.8(6.7) |  |
| Alderman,  2022 UK | Median age of  the study  population | 61 | 82(53-94) | 27(45%) | 8(2–79) | NA | 61(100%) | NA |  |
| Annweiler,  2021 France | Study population included, age >70 y | 353 | 84.7(7.0) | 193(54.7%) | NA | NA | NA | NA |  |
| Bavaro,  2021 Italy | Study population included, age >65 y | 206 | 80(72 - 86) | 108(52%) | 22(12– 39) | NA | NA | NA |  |
| Benussi^a^,  2020 Italy | Mean age  of the study population  > 65 y | 56 | 77.0(67.0–83.8) | 28(50.0%) | 6.0(3.3–10.0) | NA | NA | NA |  |
| Bianchetti,  2020 Italy | Study population included, age >65 y | 82 | 82.6(5.3) | 47(57.3%) | NA | NA | NA | NA |  |
| D'ardes,  2021 Italy | Mean age  of the study population  > 65 y | 56 | NA | 37(66%) | NA | NA | NA | 84.29(7.45) |  |
| Davis,  2021 UK | Older adults as per study population,  median age > 65 | 222 | 82(56-99) | 148(67%) | 35.35 | NA | NA | NA |  |
| De Smet,  2020 Belgium | Older adults admitted to  geriatric wards | 81 | 85(65-97) | 48(59%) | 13(8-18.5) | NA | 8(9.8%) | NA |  |
| Di Giorgio,  2022 Italy | Mean age  of the study population  > 65 y | 214 | 67.88(15.05) | 96(44.9%) | NA | 33.44(12.63) | 29(13.55%) | 80.89(8.89) |  |
| Emmerton,  2020 UK | Mean age  of the study population  > 65 y | 71 | NA | 30(42%) | NA | NA | 5(6.4%) | 70–90 |  |
| Fan,  2020 China | Mean age  of the study population  > 65 y | 86 | 66.6(11.1) | 32(37.2%) | 35.0(20.6-43.5) | NA | NA | NA |  |
| Fernández-Jiménez,  2021 Spain | Mean age  of the study population  > 65 y | 1785 | 66.8(16.6) | 798(44.7%) | NA | NA | NA | NA |  |
| Forget,  2021 Canada | Study population included, age >65 y | 127 | 82(74–88) | 54(43%) | NA | 14 (7–24) | 53 (42%) | NA |  |
| Gan,  2020 UK | Study population included, age >65 y | 122 | 81(8) | 60(49%) | NA | NA | NA | NA |  |
| Garcez,  2020 Brazil | Study population included, age >65 y | 707 | 66(11) | 303(43%) | 11(6–16) | 13 (8–20) | 129 (55%) | 70(11) |  |
| Garcia Clemente, 2020 Spain | Mean age  of the study population  > 65 y | 249 | 65.5(16.1) | 106(42.6%) | 12.1(7.5) | NA | NA | NA |  |
| GeMRC,  2021 UK/other | Mean age  of the study population  > 65 y | 5,711 | 74(54–83) | 2558(44.9%) | 8(4–16) | NA | NA | NA |  |
| Gholi,  2022 Iran | Study population included, age >65 y | 310 | 73(7) | 127(41%) | 14(10–19) | NA | NA | NA |  |
| Giorgianni,  2021 Belgium | Mean age  of the study population  > 65 y | 26 | 70.6 (21–88) | 14(53.9%) | NA | NA | NA | NA |  |
| Goldberg^b^,  2022 USA | Mean age  of the study population  > 65 y | 1375 | NA | 670(49%) | NA | NA | NA | NA |  |
| Graham^c^,  2020 UK | Older adults living in nursing homes | 126 | NA | NA | NA | NA | NA | NA |  |
| Heath,  2020 UK | Mean age  of the study population  > 65 y | 31 | 84 (76-89) | 7(22%) | NA | NA | NA | NA |  |
| Hetherington, 2020 UK | Mean age  of the study population  > 65 y | 186 | 76(71.84) | 88(47.3%) | NA | NA | NA | NA |  |
| Jäckel^a^,  2020 Germany | Mean age  of the study population  > 65 y | 20 | 65.48(10.99) | 4(20.0%) | NA | 2.8(2.4) | 2 (10.0%) | NA |  |
| Kandori,  2020 Japan | Mean age  of the study population  > 65 y | 6264 | 74(56–83) | 2961(47.3%) | NA | NA | NA | NA |  |
| Karlsson,  2021  Netherland | Study population included, age > 80 y | 102 | 84(82–88) | 54(53%) | 7.0 (4.0–11) | NA | 15(50%) | NA |  |
| Kennedy,  2020 UK | Study population included, age >65 y | 817 | 77.7 (8.2) | 431(53%) | NA | NA | 84(37%) | NA |  |
| Knights,  2020 UK | Mean age  of the study population  > 65 y | 108 | 68.7(1.5) | 45(42%) | 8(4-11) | NA | NA | NA |  |
| Knopp,  2020 UK | Study population included, age > 70 y | 217 | 80(6.8) | 83(38%) | NA | NA | NA | NA |  |
| Koduri,  2021 UK | Mean age  of the study population  > 65 y | 500 | 69.4(17.2) | 200(40%) | 9.3 (12.17) | NA | 23(48.9%) | NA |  |
| Kotfis,  2021 Poland | Mean age  of the study population  > 65 y | 201 | 68.14(13.82) | 101(50.25) | NA | 11.46(9.10) | 18 (46.15%) | 77.59(10.13) |  |
| Kremer,  2020 France | Mean age  of the study population  > 65 y | 64 | 66(20–92) | 21(33%) | NA | NA | NA | NA |  |
| Kroon,  2022 Netherland | Study population included, age > 70 y | 412 | 76(68–82) | 174(42.2%) | NA | 8 (5–13) | 37 (45.1%) | 82(77–86) |  |
| Lombardi,  2020 Italy | Mean age  of the study population  > 65 y | 614 | 67(13) | 179(29.2%) | 13 (8-23) | NA | NA | NA |  |
| Lovell,  2020 UK | Mean age  of the study population  > 65 y | 101 | 82(72-89) | 37(36%) | NA | NA | NA | NA |  |
| Maguire,  2020 UK | More than 88% of the study population aged > 70 Y | 224 | NA | 101(45%) | NA | NA | NA | NA |  |
| Marengoni,  2020 Italy | Study population included, age > 70 y | 91 | 79.5 (6.1) | 36(39.6%) | NA | NA | 18 (72.0%) | 81.7(5.5) |  |
| Martín‑Sánchez^b^, 2020 Spain | Subset of patients with age > 65 | 636 | NA | 291(45.7%) | NA | NA | NA | NA |  |
| Mattace-Raso, 2020 Netherlands | Study population included, age > 60 y | 123 | NA | 35(28%) | NA | NA | NA | 71.3 |  |
| Mendes,  2021  Switzerland | Study population included, age > 65 y | 235 | 86.3 (6.5) | 133(56.6%) | 12.8(7.6) | 12.2(6.7) | 20(41.6%) | 86.9(5.6) |  |
| Morandi,  2022 Italy, Spain | Study population included, age > 65 y | 158 | 82 (81 -83) | 71(44.9%) | NA | NA | NA | NA |  |
| Myrstad,  2020 Norway | Mean age  of the study population  > 65 y | 66 | 71.5 (30–95) | 28(42%) | NA | NA | NA | NA |  |
| Pandurangan, 2021 India | Study population included, age > 60 y | 169 | 68(7.9) | 68(40.2%) | 10 | NA | NA | NA |  |
| Parrotta,  2023 Italy | Study population included, age > 60 y | 337 | 77.1(9.5) | 169(50.1%) | NA | NA | NA | 84.2(7.8) |  |
| Patel^c^,  2020 USA | Older adults admitted to  geriatric wards | 35 | 82(75–92) | 24(69%) | NA | NA | NA | NA |  |
| Pilotto^a^,  2021 Italy | Older adults admitted to  geriatric wards | 147 | 73.1(12.4) | 75(51.0%) | NA | NA | NA | NA |  |
| Pisaturo^d^,  2021 Italy | Mean age  of the study population  > 65 y | 69 | NA | 27(39%) | NA | NA | NA | NA |  |
| Poco,  2021 Brazil | Mean age  of the study population  > 65 y | 711 | 66(11) | 305(43%) | 11 | NA | NA | NA |  |
| Poloni,  2020 Italy | Study population included, age > 65 y | 57 | 82.3(6.8) | 38(66.7) | NA | NA | 11(78.6%) | 85.4(5) |  |
| Rawle (1),  2020 UK | Study population included, age > 80 y | 70 | 85(6) | 31(44.%) | 8 | NA | NA | NA |  |
| Rawle (2),  2020 UK | Study population included, age > 80 y | 64 | 88.5(7) | 30(46.9%) | NA | NA | NA | NA |  |
| Rebora,  2021 Italy | Study population included, age > 65 y | 516 | 78(73- 84) | 198(38%) | NA | NA | NA | 84(79 -88) |  |
| Romero-Sanchez,  2020 Spain | Mean age  of the study population  > 65 y | 841 | 66.42 (14.96) | 368(43.8%) | NA | NA | NA | NA |  |
| Roxby^c^,  2020 USA | Older adults living in assisting living facilities | 80 | 85.8 (7.6) | 62(78%) | NA | NA | NA | NA |  |
| Rutten,  2020 Netherlands | Study population included, age > 65 y | 1538 | 84(9.8) | 984(63.9%) | NA | NA | NA | NA |  |
| Sacco^c^,  2020 France | Study population included, age > 65 y | 41 | 88.8(7.0) | 26(63%) | NA | NA | NA | NA |  |
| Shi^a^,  2020 USA | Study population included, age > 65 y | 146 | 85(9.3) | 80(55.9%) | NA | NA | NA | NA |  |
| Steinmeyer, 2020 France | Older adults admitted to  geriatric wards | 94 | 85.5(7.5) | 52(55.3%) | 12.0(5.5) | NA | NA | NA |  |
| Strang (1),  2020 Sweden | Mean age  of the study population  > 65 y | 1903 | 86.7(57–107) | 819(43%) | NA | NA | NA | NA |  |
| Strang (2),  2020 Sweden | Mean age  of the study population  > 65 y | 202 | 83.3(30–107) | 128(58%) | NA | NA | NA | NA |  |
| Ticinesi,  2020  Italy | Mean age  of the study population  > 65 y | 852 | 73(14) | 401(47%) | NA | NA | NA | 82(78–89) |  |
| Trevisan,  2021 Italy | Study population, age > 60 y | 981 | 78.3(9.39) | 485(49.9%) | NA | NA | NA | NA |  |
| Vena,  2020 Italy | Mean age  of the study population  > 65 y | 317 | 71(60-82) | 104(32.2%) | 12.0(5-19) | NA | 22 (75.9%) | NA |  |
| Vrillon,  2020 France | Study population included, age > 85 y | 76 | 90(86–92) | 42(55.3%) | 11(7–17) | NA | NA | NA |  |
| Wong (1),  2022 Canada | Study population included, age > 65 y | 927 | 79(72.0–87.0) | 417(45%) | 11(6.0–22.0) | NA | NA | 82.0(74.0–89.0) |  |
| Wong (2),  2022 Canada | Study population included, age > 65 y | 115 | 86(78.5–91.0) | 72(62.6%) | NA | NA | NA | NA |  |
| Zazzara (1),  2020 UK | Study population included, age > 65 y | 210 | 77.9(6.83) | 82(39%) | NA | NA | NA | NA |  |
| Zazzara (2),  2020 UK | Study population included, age > 65 y | 238 | 73(5.86) | 82(34.4%) | NA | NA | NA | NA |  |
| Zerah,  2021 France | Study population included, age > 70 y | 821 | 86(7) | 473(58%) | 9(5-14) | NA | NA | NA |  |
| NOTE TO INCLUSION CRITERIA  ^a^only subset of covid positive patients; ^b^only subgroup over 65; ^c^only subset of covid residents patients (not staff memeber); ^d^combined data of cases and controls (with and without dementia).  **^‡^**Articles that included persons younger than 65 years were selected only if the mean/median age of the study population was higher than 65 years old or if data were provided according to age groups, allowing a subgroup (of adults aged > 65 years and older) data extraction possible. | | | | | | | | | |

**Supplementary Figure 1a. Funnel plot of publication bias of studies reporting data on prevalence of delirium in older adults with COVID-19**


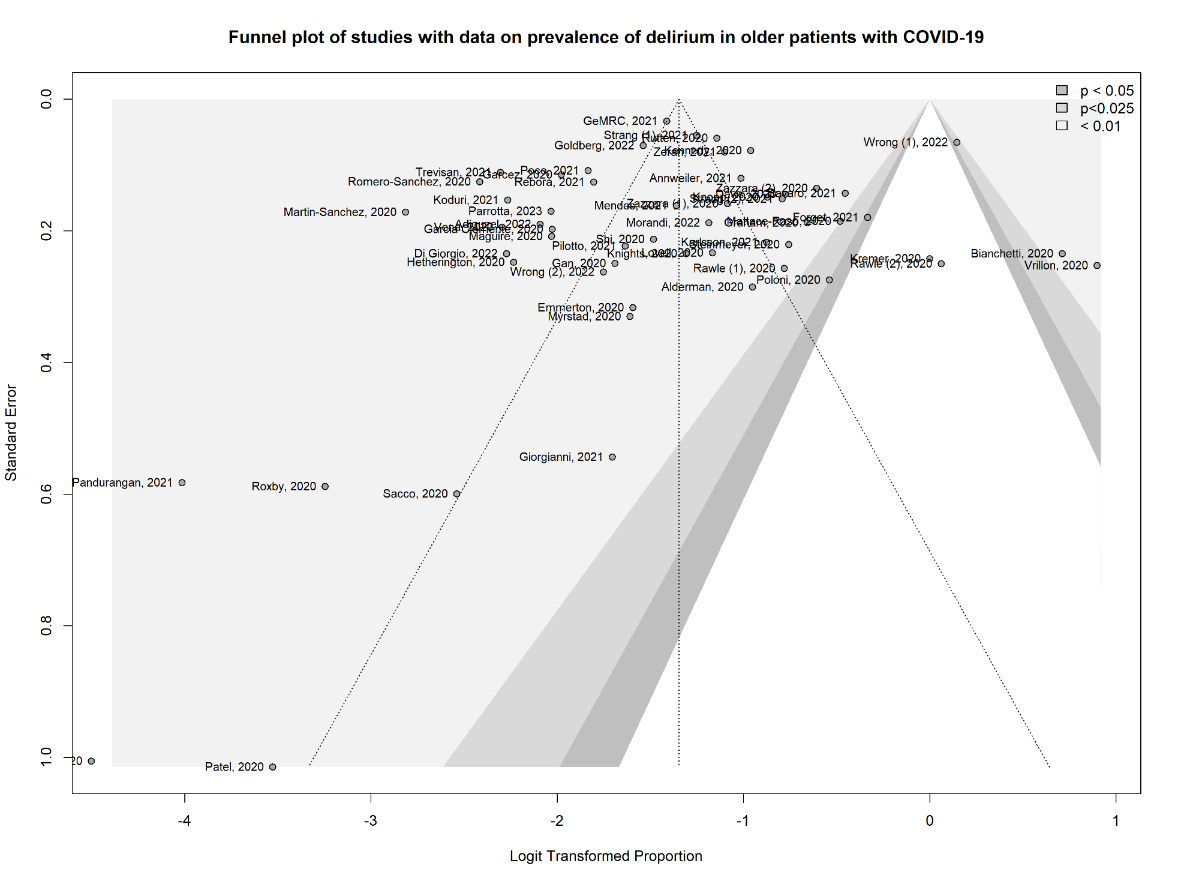


**Supplementary Figure 1b. Funnel plot of publication bias of studies reporting data on incidence of delirium in older adults with COVID-19**

**
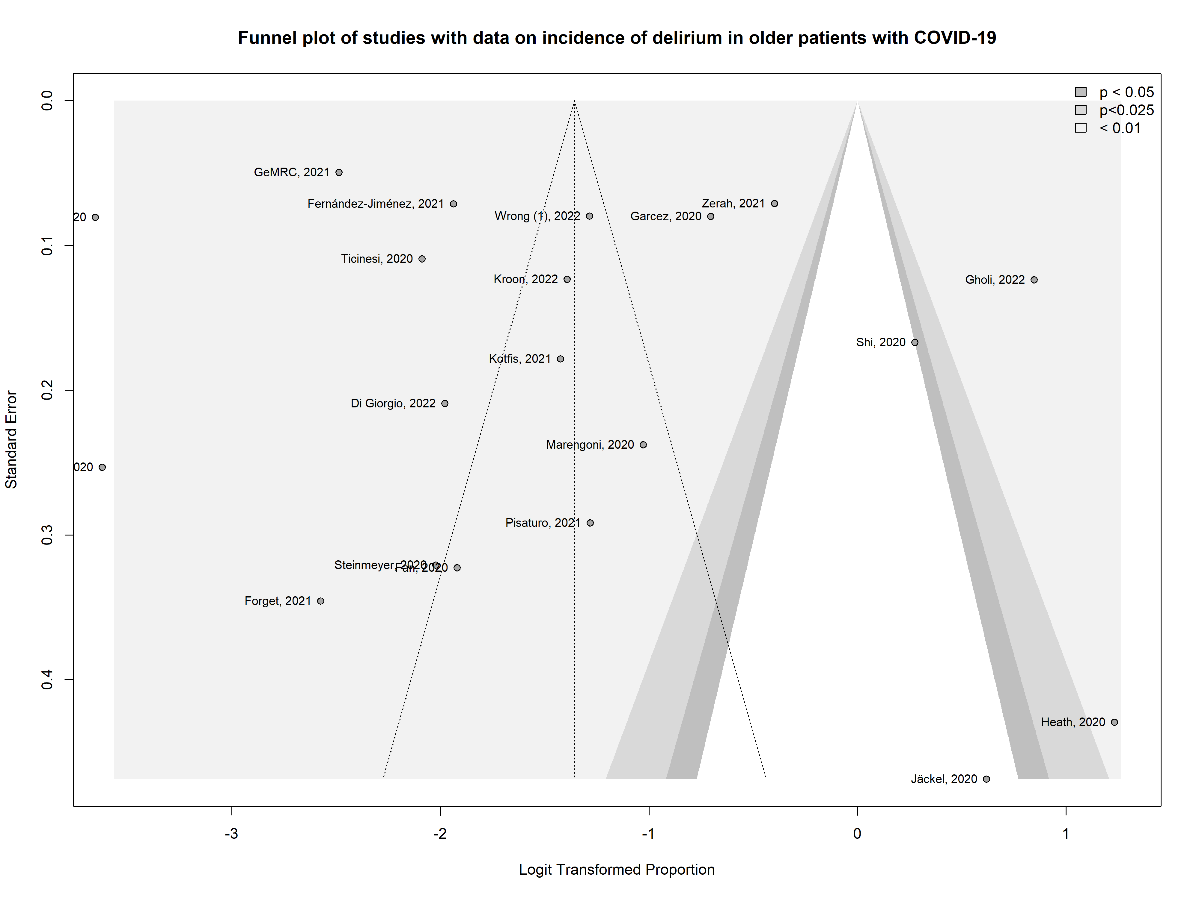
**

**Supplementary Figure 2: Forest plot of pooled occurrence of delirium in all studies.
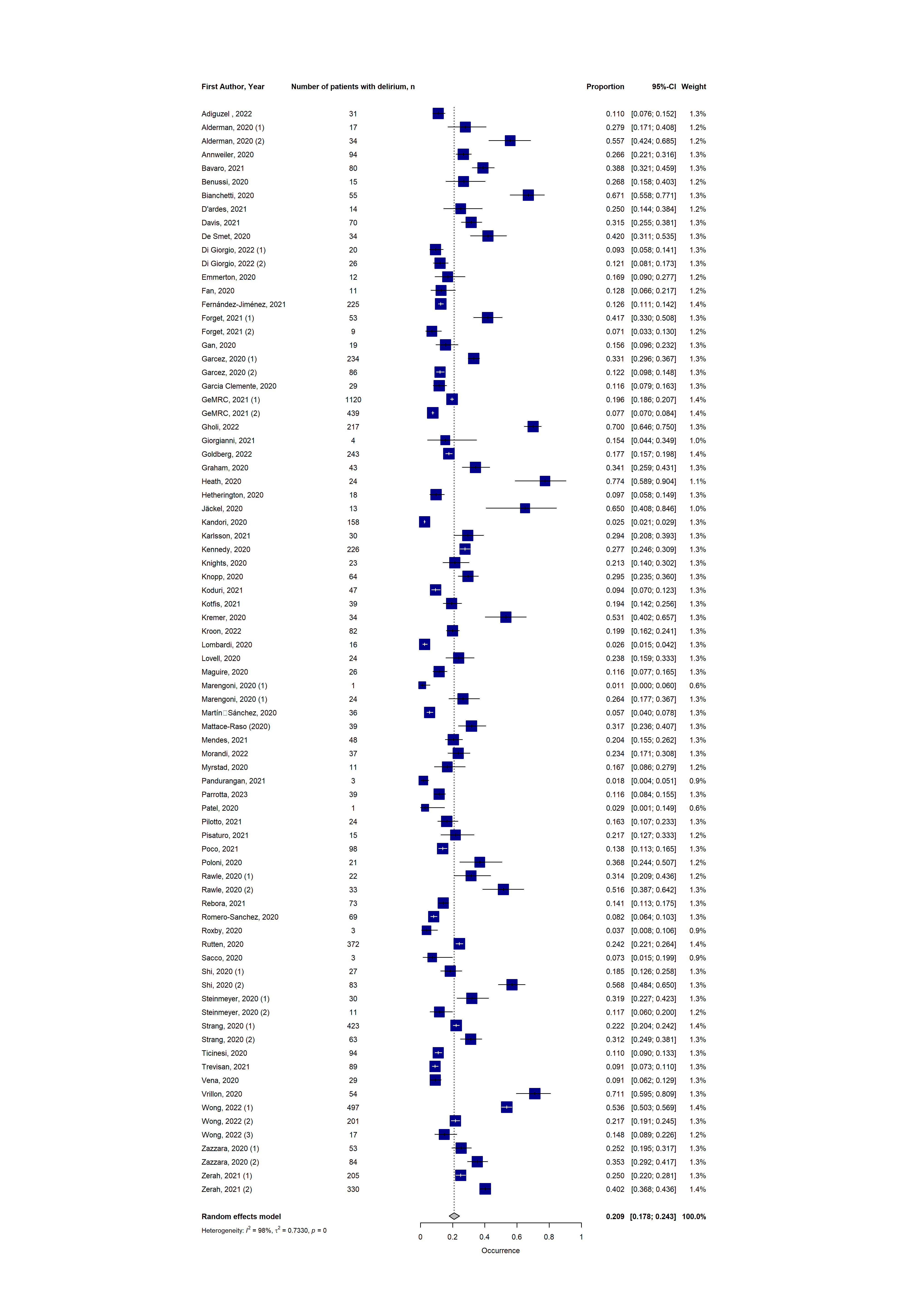
**

**Supplementary Figure 3: Forest plot of pooled occurrence of delirium according to sex.**


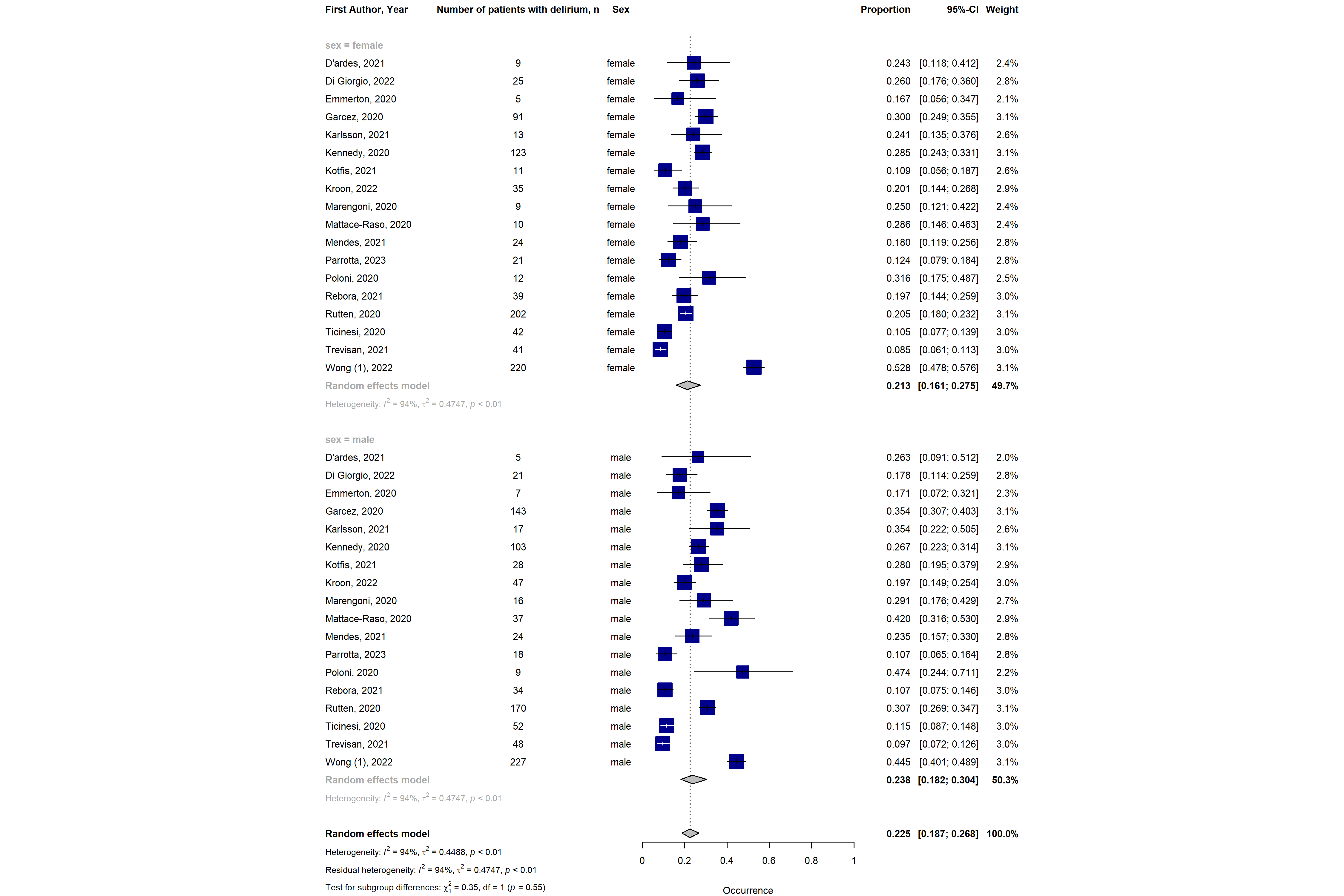


**Supplementary Figure 4:** **Forest plot of pooled occurrence of delirium according to frailty status**

**
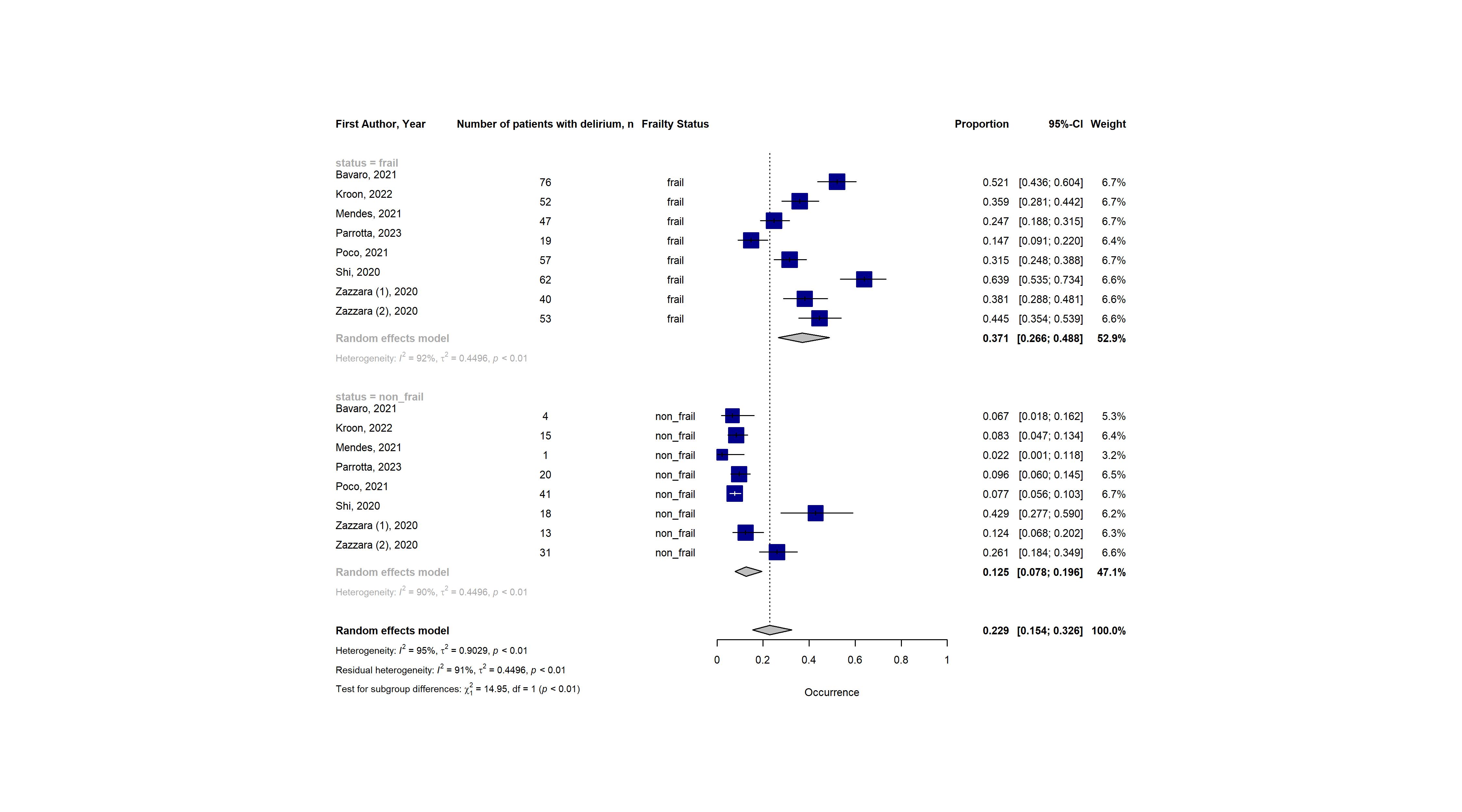
**

**Supplementary Figure 5: Occurrence of delirium according to study setting of the studies***

**
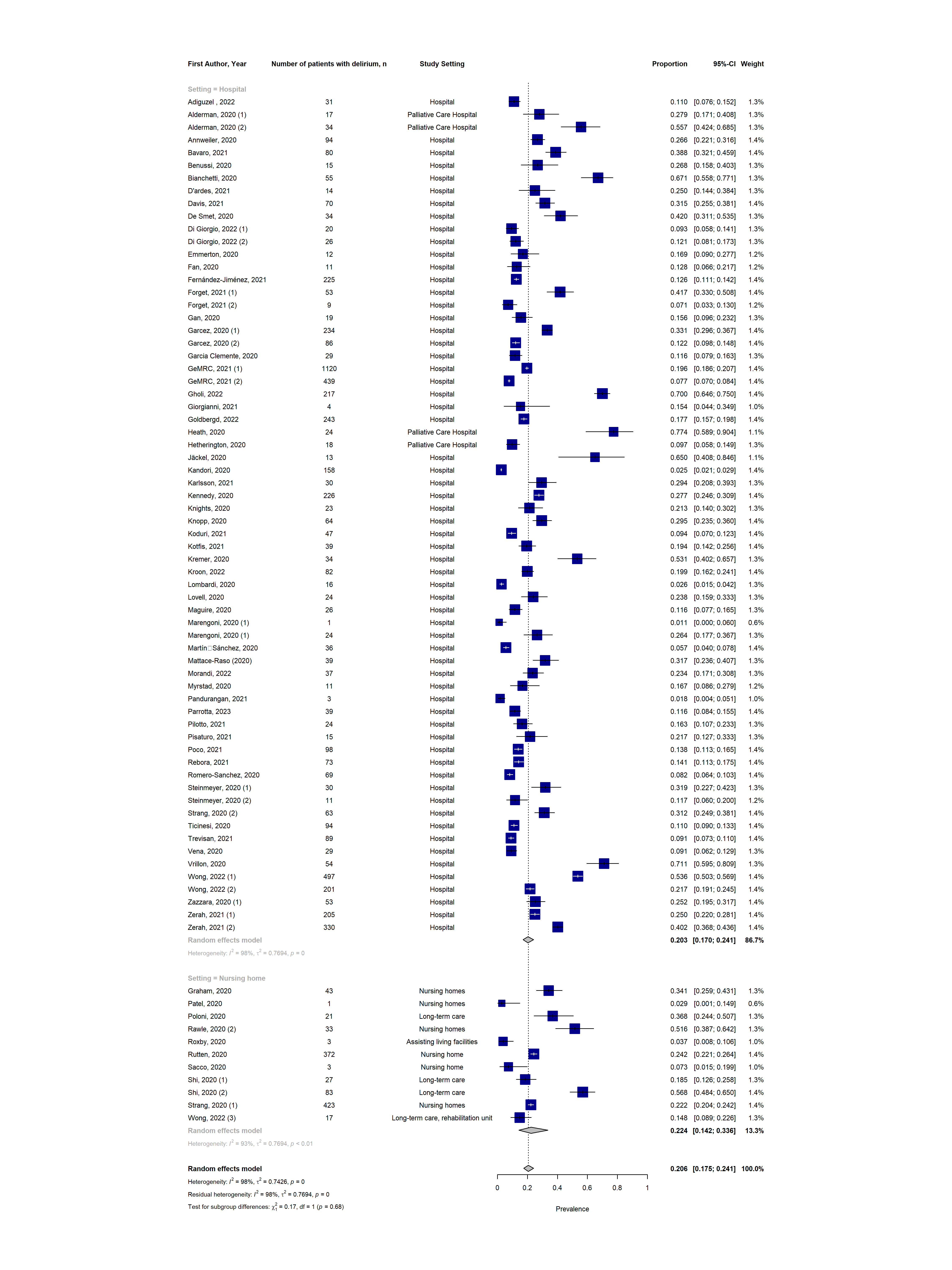
**

*Only “Hospital” and “Nursing Home” settings. Because only two studies with a total sample size of 308 observations and total number of events of 108 reported data on community-dwellers older adults affected by COVID-19, we did not include this setting in the metanalysis.

**Appendix 3**

**Prisma Check List**

| **Section and Topic** | **Item #** | **Checklist item** | **Location where item is reported** |
| --- | --- | --- | --- |
| **TITLE** | | |  |
| Title | 1 | Identify the report as a systematic review. | Page 1 |
| **ABSTRACT** | | |  |
| Abstract | 2 | See the PRISMA 2020 for Abstracts checklist. | Page 2 |
| **INTRODUCTION** | | |  |
| Rationale | 3 | Describe the rationale for the review in the context of existing knowledge. | Page 3-4 |
| Objectives | 4 | Provide an explicit statement of the objective(s) or question(s) the review addresses. | Page 4 |
| **METHODS** | | |  |
| Eligibility criteria | 5 | Specify the inclusion and exclusion criteria for the review and how studies were grouped for the syntheses. | Page 4-5 |
| Information sources | 6 | Specify all databases, registers, websites, organisations, reference lists and other sources searched or consulted to identify studies. Specify the date when each source was last searched or consulted. | Page 4 |
| Search strategy | 7 | Present the full search strategies for all databases, registers and websites, including any filters and limits used. | Appendix page 2 |
| Selection process | 8 | Specify the methods used to decide whether a study met the inclusion criteria of the review, including how many reviewers screened each record and each report retrieved, whether they worked independently, and if applicable, details of automation tools used in the process. | Page 5 |
| Data collection process | 9 | Specify the methods used to collect data from reports, including how many reviewers collected data from each report, whether they worked independently, any processes for obtaining or confirming data from study investigators, and if applicable, details of automation tools used in the process. | Page 5 |
| Data items | 10a | List and define all outcomes for which data were sought. Specify whether all results that were compatible with each outcome domain in each study were sought (e.g. for all measures, time points, analyses), and if not, the methods used to decide which results to collect. | Page 5 |
|  | 10b | List and define all other variables for which data were sought (e.g. participant and intervention characteristics, funding sources). Describe any assumptions made about any missing or unclear information. | Page 5 |
| Study risk of bias assessment | 11 | Specify the methods used to assess risk of bias in the included studies, including details of the tool(s) used, how many reviewers assessed each study and whether they worked independently, and if applicable, details of automation tools used in the process. | Page 5-6 |
| Effect measures | 12 | Specify for each outcome the effect measure(s) (e.g. risk ratio, mean difference) used in the synthesis or presentation of results. | Page 5 |
| Synthesis methods | 13a | Describe the processes used to decide which studies were eligible for each synthesis (e.g. tabulating the study intervention characteristics and comparing against the planned groups for each synthesis (item #5)). | Page 5 |
|  | 13b | Describe any methods required to prepare the data for presentation or synthesis, such as handling of missing summary statistics, or data conversions. | Page 4-5-6 |
|  | 13c | Describe any methods used to tabulate or visually display results of individual studies and syntheses. | Page 6 |
|  | 13d | Describe any methods used to synthesize results and provide a rationale for the choice(s). If meta-analysis was performed, describe the model(s), method(s) to identify the presence and extent of statistical heterogeneity, and software package(s) used. | Page 6 |
|  | 13e | Describe any methods used to explore possible causes of heterogeneity among study results (e.g. subgroup analysis, meta-regression). | Page 6 |
|  | 13f | Describe any sensitivity analyses conducted to assess robustness of the synthesized results. | NA |
| Reporting bias assessment | 14 | Describe any methods used to assess risk of bias due to missing results in a synthesis (arising from reporting biases). | NA |
| Certainty assessment | 15 | Describe any methods used to assess certainty (or confidence) in the body of evidence for an outcome. | NA |
| **RESULTS** | | |  |
| Study selection | 16a | Describe the results of the search and selection process, from the number of records identified in the search to the number of studies included in the review, ideally using a flow diagram. | Page 6 and 7, Figure 1, Appendix Supplementary Table 1a and 1bc |
|  | 16b | Cite studies that might appear to meet the inclusion criteria, but which were excluded, and explain why they were excluded. | NA |
| Study characteristics | 17 | Cite each included study and present its characteristics. | Appendix Supplementary Table 1a and 1b |
| Risk of bias in studies | 18 | Present assessments of risk of bias for each included study. | Appendix Supplementary Table 1a and 1b |
| Results of individual studies | 19 | For all outcomes, present, for each study: (a) summary statistics for each group (where appropriate) and (b) an effect estimate and its precision (e.g. confidence/credible interval), ideally using structured tables or plots. | Appendix Supplementary Table 1a and 1b  Page 6 |
| Results of syntheses | 20a | For each synthesis, briefly summarise the characteristics and risk of bias among contributing studies. | Appendix Supplementary Table 1a and 1b |
|  | 20b | Present results of all statistical syntheses conducted. If meta-analysis was done, present for each the summary estimate and its precision (e.g. confidence/credible interval) and measures of statistical heterogeneity. If comparing groups, describe the direction of the effect. | Page 6-7-8 |
|  | 20c | Present results of all investigations of possible causes of heterogeneity among study results. | NA |
|  | 20d | Present results of all sensitivity analyses conducted to assess the robustness of the synthesized results. | NA |
| Reporting biases | 21 | Present assessments of risk of bias due to missing results (arising from reporting biases) for each synthesis assessed. | NA |
| Certainty of evidence | 22 | Present assessments of certainty (or confidence) in the body of evidence for each outcome assessed. | NA |
| **DISCUSSION** | | |  |
| Discussion | 23a | Provide a general interpretation of the results in the context of other evidence. | Page 8-9 |
|  | 23b | Discuss any limitations of the evidence included in the review. | Page 10 |
|  | 23c | Discuss any limitations of the review processes used. | Page 10 |
|  | 23d | Discuss implications of the results for practice, policy, and future research. | Page 8-9-10 |
| **OTHER INFORMATION** | | |  |
| Registration and protocol | 24a | Provide registration information for the review, including register name and registration number, or state that the review was not registered. | Page 4 |
|  | 24b | Indicate where the review protocol can be accessed, or state that a protocol was not prepared. | NA |
|  | 24c | Describe and explain any amendments to information provided at registration or in the protocol. | NA |
| Support | 25 | Describe sources of financial or non-financial support for the review, and the role of the funders or sponsors in the review. | Page 12 |
| Competing interests | 26 | Declare any competing interests of review authors. | Page 12 |
| Availability of data, code and other materials | 27 | Report which of the following are publicly available and where they can be found: template data collection forms; data extracted from included studies; data used for all analyses; analytic code; any other materials used in the review. | All data available in appendix. For the code the R code package used is cited |

*From:*  Page MJ, McKenzie JE, Bossuyt PM, Boutron I, Hoffmann TC, Mulrow CD, et al. The PRISMA 2020 statement: an updated guideline for reporting systematic reviews. BMJ 2021;372:n71. doi: 10.1136/bmj.n71

For more information, visit: <http://www.prisma-statement.org/>

**Appendix 3**

**Reference List of Included Articles**

1. Adiguzel A, Ozturk U. Neurological Evaluation of Geriatric Patients Being Treated for COVID-19. *Eurasian J Med*. 2022;54(1):61-66. doi:10.5152/eurasianjmed.2022.21099

2. Alderman B, Webber K, Davies A. An audit of end-of-life symptom control in patients with corona virus disease 2019 (COVID-19) dying in a hospital in the United Kingdom. *Palliat Med*. 2020;34(9):1249-1255. doi:10.1177/0269216320947312

3. Annweiler C, Sacco G, Salles N, et al. National French Survey of Coronavirus Disease (COVID-19) Symptoms in People Aged 70 and over. *Clin Infect Dis*. 2021;72(3):490-494. doi:10.1093/cid/ciaa792

4. Bavaro DF, Diella L, Fabrizio C, et al. Peculiar clinical presentation of COVID-19 and predictors of mortality in the elderly: A multicentre retrospective cohort study. *Int J Infect Dis*. 2021;105:709-715. doi:10.1016/j.ijid.2021.03.021

5. Benussi A, Pilotto A, Premi E, et al. Clinical characteristics and outcomes of inpatients with neurologic disease and COVID-19 in Brescia, Lombardy, Italy. *Neurology*. 2020;95(7). doi:10.1212/WNL.0000000000009848

6. Bianchetti A, Rozzini R, Guerini F, et al. Clinical Presentation of COVID19 in Dementia Patients. *J Nutr Heal Aging*. 2020;24(6):560-562. doi:10.1007/s12603-020-1389-1

7. D’Ardes D, Carrarini C, Russo M, et al. Low molecular weight heparin in COVID-19 patients prevents delirium and shortens hospitalization. *Neurol Sci*. 2021;42(4):1527-1530.

8. Davis P, Gibson R, Wright E, et al. Atypical presentations in the hospitalised older adult testing positive for SARS-CoV-2: a retrospective observational study in Glasgow, Scotland. *Scott Med J*. 2021;66(2):89-97. doi:10.1177/0036933020962891

9. Smet R De, Mellaerts B, Vandewinckele H. Since January 2020 Elsevier has created a COVID-19 resource centre with free information in English and Mandarin on the novel coronavirus COVID- 19 . The COVID-19 resource centre is hosted on Elsevier Connect , the company ’ s public news and information . 2020;(January).

10. Di Giorgio A, Mirijello A, De Gennaro C, et al. Factors Associated with Delirium in COVID-19 Patients and Their Outcome: A Single-Center Cohort Study. *Diagnostics*. 2022;12(2):1-12. doi:10.3390/diagnostics12020544

11. Emmerton D, Abdelhafiz A. Delirium in older people with COVID-19: clinical scenario and literature review. *SN Compr Clin Med*. 2020;2(10):1790-1797.

12. Fan H, Tang X, Song Y, Liu P, Chen Y. Influence of COVID-19 on cerebrovascular disease and its possible mechanism. *Neuropsychiatr Dis Treat*. 2020;16:1359.

13. Fernández-Jiménez E, Muñoz-Sanjose A, Mediavilla R, et al. Prospective Analysis Between Neutrophil-to-Lymphocyte Ratio on Admission and Development of Delirium Among Older Hospitalized Patients With COVID-19. *Front Aging Neurosci*. 2021;13(November):1-6. doi:10.3389/fnagi.2021.764334

14. Forget MF, Del Degan S, Leblanc J, et al. Delirium and inflammation in older adults hospitalized for covid-19: A cohort study. *Clin Interv Aging*. 2021;16:1223-1230. doi:10.2147/CIA.S315405

15. Gan JM, Kho J, Akhunbay-Fudge M, et al. Atypical presentation of COVID-19 in hospitalised older adults. *Ir J Med Sci*. 2021;190(2):469-474. doi:10.1007/s11845-020-02372-7

16. Garcez FB, Aliberti MJR, Poco PCE, et al. Delirium and Adverse Outcomes in Hospitalized Patients with COVID-19. *J Am Geriatr Soc*. 2020;68(11):2440-2446. doi:10.1111/jgs.16803

17. García Clemente MM, Herrero Huertas J, Fernández Fernández A, et al. Assessment of risk scores in Covid-19. *Int J Clin Pract*. 2021;75(12). doi:10.1111/ijcp.13705

18. Collaborative GMR, Collaborative C, Welch C. Age and Frailty Are Independently Associated with Increased Mortality in COVID-19: Results of an International Multi-Centre Study. *SSRN Electron J*. Published online 2020. doi:10.2139/ssrn.3709847

19. Gholi Z, Yadegarynia D, Eini-Zinab H, Vahdat Shariatpanahi Z. Vitamin D deficiency is associated with increased risk of delirium and mortality among critically Ill, elderly covid-19 patients. *Complement Ther Med*. 2022;70(July). doi:10.1016/j.ctim.2022.102855

20. Giorgianni A, Vinacci G, Agosti E, Mercuri A, Baruzzi F. Neuroradiological features in COVID-19 patients: First evidence in a complex scenario. *J Neuroradiol*. 2020;47(6):474-476. doi:10.1016/j.neurad.2020.05.005

21. Goldberg EM, Southerland LT, Meltzer AC, et al. Age-related differences in symptoms in older emergency department patients with COVID-19: Prevalence and outcomes in a multicenter cohort. *J Am Geriatr Soc*. 2022;70(7):1918-1930. doi:10.1111/jgs.17816

22. Graham NSN, Junghans C, Downes R, et al. SARS-CoV-2 infection, clinical features and outcome of COVID-19 in United Kingdom nursing homes. *J Infect*. 2020;81(3):411-419. doi:10.1016/j.jinf.2020.05.073

23. Heath L, Yates S, Carey M, Miller M. Palliative Care During COVID-19: Data and Visits From Loved Ones. *Am J Hosp Palliat Med*. 2020;37(11):988-991. doi:10.1177/1049909120943577

24. Hetherington L, Johnston B, Kotronoulas G, Finlay F, Keeley P, McKeown A. COVID-19 and Hospital Palliative Care – A service evaluation exploring the symptoms and outcomes of 186 patients and the impact of the pandemic on specialist Hospital Palliative Care. *Palliat Med*. 2020;34(9):1256-1262. doi:10.1177/0269216320949786

25. Jäckel M, Bemtgen X, Wengenmayer T, Bode C, Biever PM, Staudacher DL. Is delirium a specific complication of viral acute respiratory distress syndrome? *Crit Care*. 2020;24(1):1-4. doi:10.1186/s13054-020-03136-6

26. Kandori K, Okada Y, Ishii W, Narumiya H, Maebayashi Y, Iizuka R. Association between visitation restriction during the COVID-19 pandemic and delirium incidence among emergency admission patients: a single-center retrospective observational cohort study in Japan. *J Intensive Care*. 2020;8(1):1-9.

27. Karlsson LK, Jakobsen LH, Hollensberg L, et al. Clinical presentation and mortality in hospitalized patients aged 80+years with COVID-19-A retrospective cohort study. *Arch Gerontol Geriatr*. 2021;94. doi:10.1016/j.archger.2020.104335

28. Kennedy M, Helfand BKI, Gou RY, et al. Delirium in Older Patients with COVID-19 Presenting to the Emergency Department. *JAMA Netw Open*. 2020;3(11):1-12. doi:10.1001/jamanetworkopen.2020.29540

29. Knights H, Mayor N, Millar K, et al. Characteristics and outcomes of patients with COVID-19 at a district general hospital in Surrey, UK. *Clin Med J R Coll Physicians London*. 2020;20(5):E148-E153. doi:10.7861/CLINMED.2020-0303

30. Knopp P, Miles A, Webb TE, et al. Presenting features of COVID-19 in older people: relationships with frailty, inflammation and mortality. *Eur Geriatr Med*. 2020;11(6):1089-1094. doi:10.1007/s41999-020-00373-4

31. Koduri G, Gokaraju S, Darda M, et al. Clinical frailty score as an independent predictor of outcome in COVID-19 hospitalised patients. *Eur Geriatr Med*. 2021;12(5):1065-1073. doi:10.1007/s41999-021-00508-1

32. Kotfis K, Roberson SW, Wilson J, et al. COVID-19: What do we need to know about ICU delirium during the SARS-CoV-2 pandemic? *Anaesthesiol Intensive Ther*. 2020;52(2):132-138.

33. Kremer S, Lersy F, Anheim M, et al. Neurologic and neuroimaging findings in patients with COVID-19: A retrospective multicenter study. *Neurology*. 2020;95(13):E1868-E1882. doi:10.1212/WNL.0000000000010112

34. Kroon B, Beishuizen SJE, van Rensen IHT, et al. Delirium in older COVID-19 patients: Evaluating risk factors and outcomes. *Int J Geriatr Psychiatry*. 2022;37(10). doi:10.1002/gps.5810

35. Lombardi CM, Carubelli V, Iorio A, et al. Association of Troponin Levels With Mortality in Italian Patients Hospitalized With Coronavirus Disease 2019: Results of a Multicenter Study. *JAMA Cardiol*. 2020;5(11):1274-1280. doi:10.1001/jamacardio.2020.3538

36. Lovell N, Maddocks M, Etkind SN, et al. Characteristics, Symptom Management, and Outcomes of 101 Patients With COVID-19 Referred for Hospital Palliative Care. *J Pain Symptom Manage*. 2020;60(1):e77-e81. doi:10.1016/j.jpainsymman.2020.04.015

37. Maguire D, Woods M, Richards C, et al. Prognostic factors in patients admitted to an urban teaching hospital with COVID-19 infection. *J Transl Med*. 2020;18(1):1-10. doi:10.1186/s12967-020-02524-4

38. Marengoni A, Zucchelli A, Grande G, Fratiglioni L, Rizzuto D. The impact of delirium on outcomes for older adults hospitalised with COVID--19. *Age Ageing*. 2020;49(6):923-926. doi:10.1093/ageing/afaa189

39. Martín-Sánchez FJ, del Toro E, Cardassay E, et al. Clinical presentation and outcome across age categories among patients with COVID-19 admitted to a Spanish Emergency Department. *Eur Geriatr Med*. 2020;11(5):829-841. doi:10.1007/s41999-020-00359-2

40. Mattace-Raso F, Polinder-Bos H, Oosterwijk B, et al. Delirium: a frequent manifestation in COVID-19 older patients. *Clin Interv Aging*. 2020;15:2245.

41. Mendes A, Herrmann FR, Perivier S, Gold G, Graf CE, Zekry D. Delirium in Older Patients With COVID-19: Prevalence, Risk Factors, and Clinical Relevance. *Journals Gerontol Ser a-Biological Sci Med Sci*. 2021;76(8):E142-E146. doi:10.1093/gerona/glab039

42. Morandi A, Gual N, Cesari M, et al. Geriatric syndromes and functions in older adults with COVID-19 hospitalized in sub-acute care: a multicenter study. *Aging Clin Exp Res*. doi:10.1007/s40520-022-02264-z

43. Myrstad M, Ihle-Hansen H, Tveita AA, et al. National Early Warning Score 2 (NEWS2) on admission predicts severe disease and in-hospital mortality from Covid-19 - A prospective cohort study. *Scand J Trauma Resusc Emerg Med*. 2020;28(1):1-8. doi:10.1186/s13049-020-00764-3

44. Pandurangan V, Gopalan S, Madhavan S, et al. Hospitalization Outcomes and Mortality Predictors of Sars-Cov-2 Infection in the Elderly: A Single Center Experience from India. *Arch Clin Infect Dis*. 2021;16(2). doi:10.5812/archcid.112397

45. Parrotta I, Bencivenga L, Okoye C, et al. Frailty and hyperactive delirium in hospitalized older patients with COVID-19: an insight from GeroCovid registry. *Aging Clin Exp Res*. 2023;(0123456789). doi:10.1007/s40520-022-02328-0

46. Patel MC, Chaisson LH, Borgetti S, et al. Asymptomatic SARS-CoV-2 infection and COVID-19 mortality during an outbreak investigation in a skilled nursing facility. *Clin Infect Dis*. 2020;71(11):2920-2926. doi:10.1093/cid/ciaa763

47. Pilotto A, Benussi A, Libri I, et al. COVID-19 impact on consecutive neurological patients admitted to the emergency department. *J Neurol Neurosurg Psychiatry*. 2021;92(2):218-220. doi:10.1136/jnnp-2020-323929

48. Pisaturo M, Calo F, Russo A, et al. Dementia as Risk Factor for Severe Coronavirus Disease 2019: A Case-Control Study. *Front Aging Neurosci*. 2021;13. doi:10.3389/fnagi.2021.698184

49. Poco PCE, Aliberti MJR, DIas MB, et al. Divergent: Age, Frailty, and Atypical Presentations of COVID-19 in Hospitalized Patients. *Journals Gerontol - Ser A Biol Sci Med Sci*. 2021;76(3):E46-E51. doi:10.1093/gerona/glaa280

50. Poloni TE, Carlos AF, Cairati M, et al. Prevalence and prognostic value of Delirium as the initial presentation of COVID-19 in the elderly with dementia: An Italian retrospective study. *EClinicalMedicine*. 2020;26:100490. doi:10.1016/j.eclinm.2020.100490

51. Rawle MJ, Bertfield DL, Brill SE. Atypical presentations of COVID-19 in care home residents presenting to secondary care: A UK single centre study. *Aging Med*. 2020;3(4):237-244. doi:10.1002/agm2.12126

52. Rebora P, Rozzini R, Bianchetti A, et al. Delirium in Patients with SARS-CoV-2 Infection: A Multicenter Study. *J Am Geriatr Soc*. 2021;69(2):293-299. doi:10.1111/jgs.16969

53. Romero-Sánchez CM, Díaz-Maroto I, Fernández-Díaz E, et al. Neurologic manifestations in hospitalized patients with COVID-19: The ALBACOVID registry. *Neurology*. 2020;95(8):E1060-E1070. doi:10.1212/WNL.0000000000009937

54. Roxby AC, Greninger AL, Hatfield KM, et al. Outbreak investigation of COVID-19 among residents and staff of an independent and assisted living community for older adults in Seattle, Washington. *JAMA Intern Med*. 2020;180(8):1101-1105. doi:10.1001/jamainternmed.2020.2233

55. Rutten JJS, van Loon AM, van Kooten J, et al. Clinical Suspicion of COVID-19 in Nursing Home Residents: Symptoms and Mortality Risk Factors. *J Am Med Dir Assoc*. 2020;21(12):1791-1797.e1. doi:10.1016/j.jamda.2020.10.034

56. Sacco G, Foucault G, Briere O, Annweiler C. Since January 2020 Elsevier has created a COVID-19 resource centre with free information in English and Mandarin on the novel coronavirus COVID- 19 . The COVID-19 resource centre is hosted on Elsevier Connect , the company ’ s public news and information . 2020;(January).

57. Shi SM, Bakaev I, Chen H, Travison TG, Berry SD. Risk Factors, Presentation, and Course of Coronavirus Disease 2019 in a Large, Academic Long-Term Care Facility. *J Am Med Dir Assoc*. 2020;21(10):1378-1383.e1. doi:10.1016/j.jamda.2020.08.027

58. Steinmeyer Z, Vienne-Noyes S, Bernard M, et al. Acute Care of Older Patients with COVID-19: Clinical Characteristics and Outcomes. *Geriatrics*. 2020;5(4). doi:10.3390/geriatrics5040065

59. Strang P, Martinsson L, Bergstrom J, Lundstrom S. COVID-19: Symptoms in Dying Residents of Nursing Homes and in Those Admitted to Hospitals. *J Palliat Med*. 2021;24(7):1067-1071. doi:10.1089/jpm.2020.0688

60. Ticinesi A, Cerundolo N, Parise A, et al. Delirium in COVID-19: epidemiology and clinical correlations in a large group of patients admitted to an academic hospital. *Aging Clin Exp Res*. 2020;32(10):2159-2166.

61. Trevisan C, Remelli F, Fumagalli S, et al. COVID-19 as a Paradigmatic Model of the Heterogeneous Disease Presentation in Older People: Data from the GeroCovid Observational Study. *Rejuvenation Res*. 2022;25(3):129-140. doi:10.1089/rej.2021.0063

62. Vena A, Giacobbe DR, Di Biagio A, et al. Clinical characteristics, management and in-hospital mortality of patients with coronavirus disease 2019 in Genoa, Italy. *Clin Microbiol Infect*. 2020;26(11):1537-1544. doi:10.1016/j.cmi.2020.07.049

63. Vrillon A, Hourregue C, Azuar J, et al. COVID-19 in Older Adults: A Series of 76 Patients Aged 85 Years and Older with COVID-19. *J Am Geriatr Soc*. 2020;68(12):2735-2743. doi:10.1111/jgs.16894

64. Wong EK-C, Watt J, Zou H, et al. Characteristics, treatment and delirium incidence of older adults hospitalized with COVID-19: a multicentre retrospective cohort study. *Can Med Assoc Open Access J*. 2022;10(3):E692-E701.

65. Zazzara MB, Penfold RS, Roberts AL, et al. Probable delirium is a presenting symptom of COVID-19 in frail, older adults: a cohort study of 322 hospitalised and 535 community-based older adults. *Age Ageing*. Published online 2020. doi:10.1093/ageing/afaa223

66. Zerah L, Baudouin É, Pépin M, et al. Clinical Characteristics and Outcomes of 821 Older Patients with SARS-Cov-2 Infection Admitted to Acute Care Geriatric Wards. *Journals Gerontol - Ser A Biol Sci Med Sci*. 2021;76(3):E4-E12. doi:10.1093/gerona/glaa210
